# HER2 drives lung fibrosis by activating a metastatic cancer signature in invasive lung fibroblasts

**DOI:** 10.1084/jem.20220126

**Published:** 2022-08-18

**Authors:** Xue Liu, Yan Geng, Jiurong Liang, Ana Lucia Coelho, Changfu Yao, Nan Deng, Yizhou Wang, Kristy Dai, Guanling Huang, Ting Xie, Ningshan Liu, Simon C. Rowan, Forough Taghavifar, Vrishika Kulur, Zhenqiu Liu, Barry R. Stripp, Cory M. Hogaboam, Dianhua Jiang, Paul W. Noble

**Affiliations:** 1 Department of Medicine and Women’s Guild Lung Institute, Cedars-Sinai Medical Center, Los Angeles, CA; 2 School of Pharmaceutical Science, Jiangnan University, Wuxi, Jiangsu, China; 3 Biostatistics and Bioinformatics Research Center and Samuel Oschin Comprehensive Cancer Institute, Los Angeles, CA; 4 Genomics Core, Cedars-Sinai Medical Center, Los Angeles, CA; 5 Department of Biomedical Sciences, Cedars-Sinai Medical Center, Los Angeles, CA

## Abstract

Progressive tissue fibrosis, including idiopathic pulmonary fibrosis (IPF), is characterized by excessive recruitment of fibroblasts to sites of tissue injury and unremitting extracellular matrix deposition associated with severe morbidity and mortality. However, the molecular mechanisms that control progressive IPF have yet to be fully determined. Previous studies suggested that invasive fibroblasts drive disease progression in IPF. Here, we report profiling of invasive and noninvasive fibroblasts from IPF patients and healthy donors. Pathway analysis revealed that the activated signatures of the invasive fibroblasts, the top of which was ERBB2 (HER2), showed great similarities to those of metastatic lung adenocarcinoma cancer cells. Activation of HER2 in normal lung fibroblasts led to a more invasive genetic program and worsened fibroblast invasion and lung fibrosis, while antagonizing HER2 signaling blunted fibroblast invasion and ameliorated lung fibrosis. These findings suggest that HER2 signaling may be a key driver of fibroblast invasion and serve as an attractive target for therapeutic intervention in IPF.

## Teaser

This study demonstrates the regulatory pathways closely associated with a metastatic cancer signature regulated by HER2 in invasive IPF lung fibroblasts. Manipulation of HER2 in vitro and in vivo shows that HER2 signaling is a key driver of fibroblast invasion and progressive lung fibrosis.

## Graphical Abstract

**Figure:**
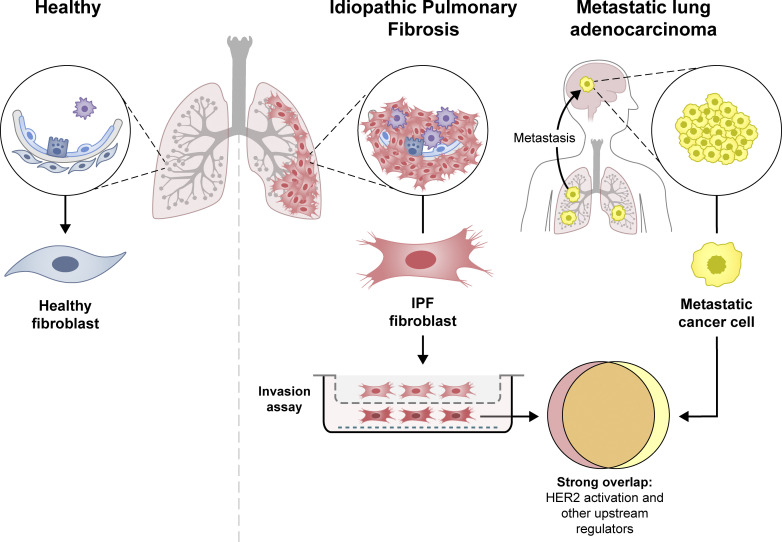

## Introduction

Progressive tissue fibrosis is a major cause of morbidity and mortality, initiated by dysregulated wound healing response to tissue injury (Noble, 2006). Idiopathic pulmonary fibrosis (IPF) is a chronic and progressive lung disease of unknown cause that leads to the destruction of the gas-exchanging regions of the lung with the accumulation of fibroblasts that produce massive extracellular matrix (ECM). Although pirfenidone and nintedanib have been approved by the FDA to slow IPF progression and both therapies have been shown to reduce the rate of decline in the forced vital capacity (King et al., 2014; Richeldi et al., 2014), neither of these agents improves lung function or reduces fibrosis, and most patients succumb or require lung transplantation within 5 yr of diagnosis. Thus, the next breakthrough needs to identify novel targets to improve IPF treatment and to develop more effective and systematic therapies.

Lung fibroblasts reside in the interstitial spaces between the alveolar and capillary basal laminae under normal conditions (Kuhn et al., 1989). Pathologic hallmarks of IPF are emergence of fibroblastic foci (King et al., 2014; Noble et al., 2012) and destruction of basement membrane (Pardo and Selman, 2012). Lung fibroblasts are heterogenous cells (Liu et al., 2021) and histological observation of the fibroblastic foci indicates that a population of fibroblasts may migrate or invade through alveolar basement membranes after lung injury (Kuhn et al., 1989). We and others demonstrated that the invasive phenotype of lung fibroblasts promotes severe fibrosis (Ahluwalia et al., 2016; Chen et al., 2016; Geng et al., 2019; Huan et al., 2015; Li et al., 2011; Lovgren et al., 2011; Xie et al., 2016). Targeting lung fibroblast invasiveness has been shown to be of therapeutic benefit in treating lung fibrosis in vivo. In a bleomycin-induced pulmonary fibrosis model, fibroblasts with Hyaluronan synthase 2 overexpression showed a higher capacity to invade matrix and promote the development of lung fibrosis (Li et al., 2011). In addition, both the invasive phenotype and progressive fibrosis were inhibited in the absence of the hyaluronan receptor, CD44 (Li et al., 2011). Fibroblasts from bleomycin-treated β-arrestin knockout mice failed to invade ECM and loss of β-arrestin resulted in protection from mortality, inhibition of matrix deposition, and preserved lung function (Lovgren et al., 2011). We recently identified that fibroblasts from IPF patients with high PDL1 expression showed greater migration and invasive capacity. In a humanized severe combined immunodeficient (SCID) IPF model, targeting PDL1 in fibroblasts by CRISPR knockout or anti-PDL1 neutralizing antibodies significantly inhibited fibroblast invasion in vitro and attenuated lung fibrosis in vivo (Geng et al., 2019). These studies suggest that invasive fibroblasts may be an attractive target to develop treatments for IPF.

To further define the molecular mechanisms of fibroblast invasion, we performed a single-cell RNA sequencing (scRNA-seq) survey of invasive and noninvasive fibroblasts from four normal and four IPF human lungs using an in vitro assay system, which had been previously used to evaluate the ability of lung fibroblasts to spontaneously invade Matrigel (Ahluwalia et al., 2016; Li et al., 2011) and commonly used to analyze the metastatic potential of cancer cells (Qian et al., 1994). In combination with our previously published bulk RNA-seq analysis (Geng et al., 2019), we comprehensively identified the gene signatures of invasive and noninvasive lung fibroblasts, and specific cell-surface markers and key transcription factors were further confirmed functionally. Ingenuity Pathway Analysis (IPA; Kramer et al., 2014) on the specific differentially expressed genes of invasive fibroblasts revealed metastatic lung adenocarcinoma-associated regulator signatures, in which the ERBB2 (HER2) signaling pathway was the most significantly activated upstream regulator. HER2 activation in normal fibroblasts dramatically instilled normal lung fibroblasts with fibroblast invasive-related gene signatures and increased fibroblast invasion and fibrosis capability, while blocking HER2 in IPF lung fibroblasts reversed invasive genetic signatures and inhibited fibroblast invasion and lung fibrosis. These data suggest that HER2 endows lung fibroblasts with a metastatic lung cancer–related signature program, which potentially defines HER2 as the master regulator of IPF lung fibroblast invasion and subsequently lung fibrosis.

## Results

### Gene expression profiles of human lung invasive fibroblasts

**Figure S1.**
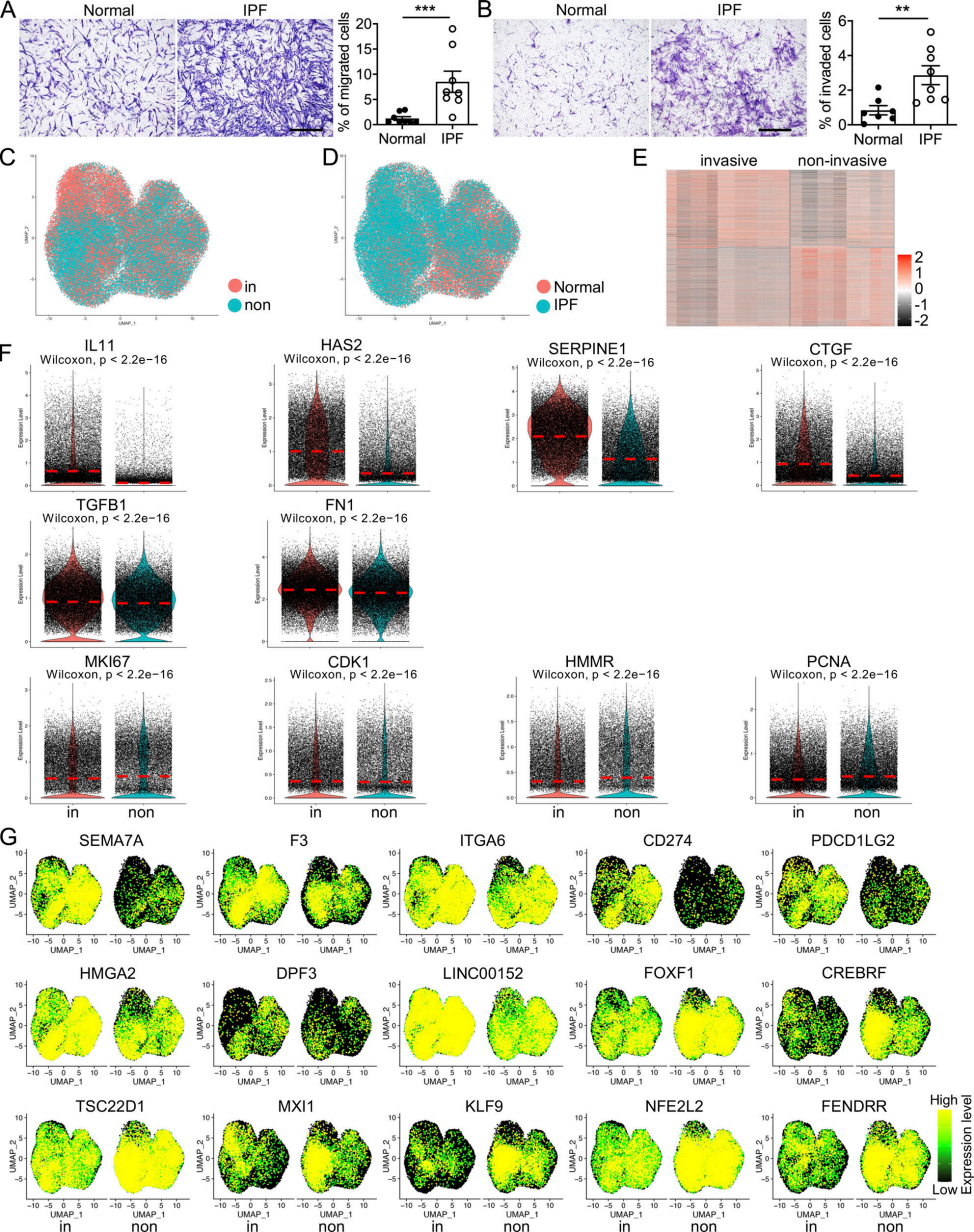
**Identification of novel marker genes of invasive and noninvasive lung fibroblasts. (A and B)** Cell migration (A) and invasion (B) assays were performed on normal and IPF fibroblasts (A, *n* = 8 per group; B, *n* = 7 for normal and *n* = 8 for IPF). **(C and D)** Visualization of the distribution of invasive and noninvasive cells (C) and healthy and IPF cells (D). **(E)** Heatmap of the top 1,000 significant genes in invasive and noninvasive fibroblasts. **(F)** Violin plot visualization of the canonical lung fibrosis related genes and cell proliferation marker genes. **(G)** Identification of the novel cell-surface marker, LincRNA, and transcriptional factor genes differentially expressed in invasive and noninvasive fibroblasts. non, noninvasive; in, invasive. Three independent experiments were performed on fibroblasts from different patients (A and B). Data are the mean ± SEM. **, P < 0.01; ***, P < 0.001 by Student’s *t* test (A and B).

Severe lung fibrosis required an invasive fibroblast phenotype, and fibroblasts from IPF lungs showed significantly increased invasive capacity compared to normal lung fibroblasts (Fig. S1, A and B). To further explore the underlying mechanisms, we investigated single cells isolated from invasive and noninvasive lung fibroblasts from four IPF patients and four healthy controls using GemCode system (10X Genomics), which is based on a high-throughput Droplet-based platform (Zheng et al., 2017) as we have previously reported with mouse lung fibroblasts (Liu et al., 2021; Xie et al., 2018). Low-quality cells were removed and retained invasive and noninvasive fibroblasts were integrated for subsequent analyses (Fig. S1, C and D; and Table S1). Differentially expressed genes between invasive and noninvasive fibroblasts were determined (Fig. S1 E). Genes, including *IL11* (Ng et al., 2019), *HAS2* (Li et al., 2011; Yang et al., 2019), *SERPINE1* (PAI-1; Chuang-Tsai et al., 2003; Loskutoff and Quigley, 2000), *CD274* (PDL1), and *PDCD1LG2* (PDL2; Geng et al., 2019), were reported to promote fibrogenesis as markers of invasive fibroblasts in fibrotic tissue and showed extremely high expression in invasive fibroblasts (Fig. S1, F and G), which validated the scRNA-seq data. Several activated fibroblast (myofibroblast)-specific genes were also upregulated, and no significant differences were identified in the expression of cell proliferating/cell cycle genes (Fig. S1 F). In addition, we determined that several genes including cell-surface protein genes, long noncoding RNA genes, and transcriptional factor genes that were differentially expressed and were potentially crucial to distinguish invasive fibroblasts from noninvasive fibroblasts (Fig. S1 G).

### Cell-surface markers distinguish invasive from noninvasive fibroblasts

**Figure 1.**
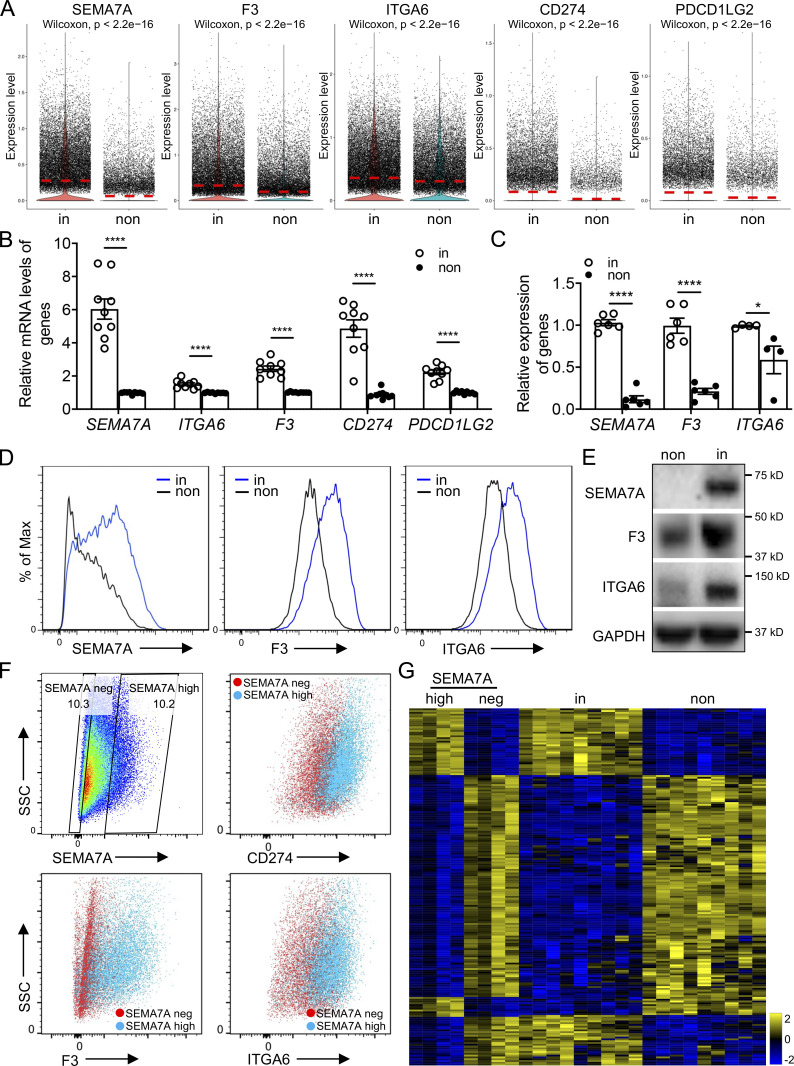
**Cell-surface markers were used to identify invasive fibroblasts. (A)** Violin plot comparisons of cell-surface marker gene expression in invasive and noninvasive fibroblasts. **(B)** Relative expression of cell-surface marker genes in bulk RNA-seq on invasive and noninvasive fibroblasts (*n* = 9 per group). **(C)** Relative expression of *SEMA7A*, *F3*, and *ITGA6* in invasive and noninvasive fibroblasts by qRT-PCR (*n* = 6 for *SEMA7A* and *F3*, and *n* = 4 for *ITGA6*). **(D)** Cell-surface expression of SEMA7A, F3, and ITGA6 in invasive and noninvasive fibroblasts by flow cytometry. **(E)** Western blot analysis of SEMA7A, F3, and ITGA6 expression in invasive and noninvasive fibroblasts. GAPDH served as loading control. **(F)** Cell-surface expression of CD274, F3, and ITGA6 in SEMA7A negative and high fibroblasts by flow cytometry. **(G)** Heatmap of consistent genes in SEMA7A^high^ and invasive, SEMA7A^negative^ and noninvasive fibroblasts, respectively, by bulk RNA-seq. non, noninvasive fibroblasts; in, invasive fibroblasts; neg, negative. Three independent experiments were performed on fibroblasts from different patients (B and C). Data are the mean ± SEM. *, P < 0.05; ****, P < 0.0001 by student’s *t* test (B and C). Source data are available for this figure: SourceData F1.

We recently reported that the cell-surface immune checkpoint ligand CD274 (also known as PDL1) was significantly upregulated on invasive lung fibroblasts and was required for the invasive phenotype of IPF fibroblasts. CD274 drove lung fibrosis in a humanized IPF model in mice (Geng et al., 2019). Here the scRNA-seq revealed that both *CD274* and *PDCD1LG2* (also known as PDL2) were upregulated in the invasive fibroblasts, which confirmed our previous observation at bulk RNA levels (Fig. 1 A and Fig. S1, F–G).

To better distinguish invasive and noninvasive lung fibroblasts, we identified several more cell-surface marker genes that showed more significant differential expression. By scRNA-seq data analysis, we found that *SEMA7A* (CD108), *F3* (CD142), and *ITGA6* (CD49f) were significantly upregulated in the invasive fibroblasts (Fig. 1 A and Fig. S1 G). As validation, the expression levels of these cell-surface protein genes were further confirmed by the published bulk RNA-seq data (Geng et al., 2019; Fig. 1 B). mRNA levels, cell surface, and total protein levels of SEMA7A, F3, and ITGA6 were, respectively, corroborated by quantitative RT-PCR (qRT-PCR; Fig. 1 C), flow cytometry (Fig. 1 D), and Western blot (Fig. 1 E). Furthermore, cell-surface expression of CD274, F3, and ITGA6 showed significant correlation with that of SEMA7A (Fig. 1 F), suggesting that these cell-surface proteins, especially SEMA7A, could be a promising cell-surface marker of invasive lung fibroblasts. To confirm this, we performed RNA-seq on flow-sorted SEMA7A^high^ and SEMA7A^negative^ fibroblasts from IPF lungs and compared the RNA-seq data on invasive and noninvasive fibroblast data we recently published (Geng et al., 2019). We found that SEMA7A^high^ and SEMA7A^negative^ cells showed similar transcriptome profiles with invasive and noninvasive fibroblasts, respectively (Fig. 1 G).

**Figure 2.**
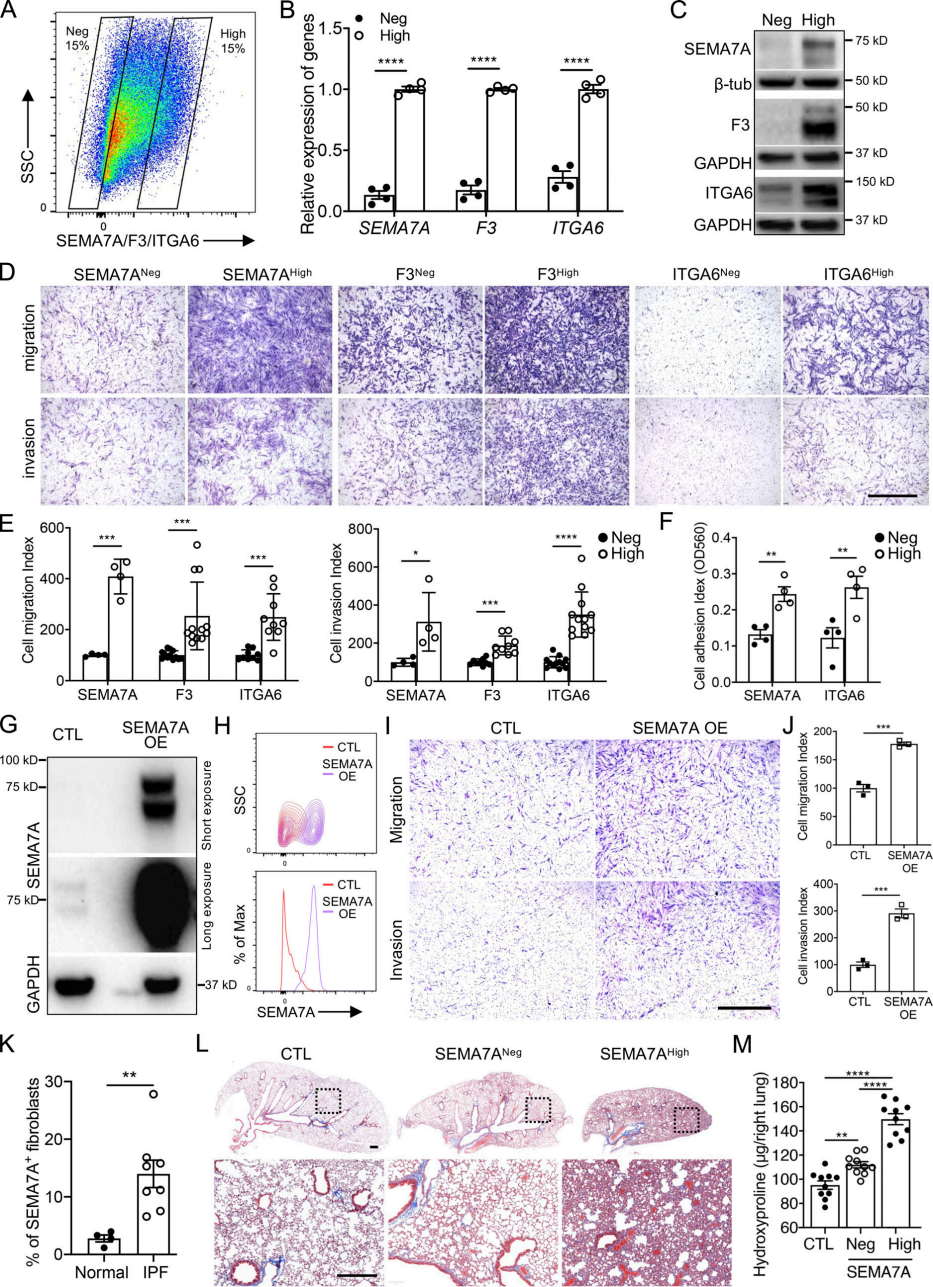
**Specific cell-surface marker genes of invasive fibroblasts promoted invasion and fibrosis. (A)** Cell sorting strategy of F3, SEMA7A, and ITGA6 negative and high fibroblasts for following experiments. **(B and C)** Relative mRNA levels (B; *n* = 4 per group) and total protein levels (C) of F3, SEMA7A, and ITGA6 expression in sorted F3, SEMA7A, and ITGA6 negative and high fibroblasts. **(D and E)** Representative images (D) and index (E; SEMA7A^Neg/High^ migration/invasion, *n* = 4 per group; F3^Neg/High^ migration, *n* = 12 per group, invasion, *n* = 9 per group; ITGA6^Neg/High^ migration, *n* = 9 per group, invasion, *n* = 12 per group) of migration and invasion of SEMA7A, F3, and ITGA6 negative and high fibroblasts. **(F)** Cell adhesion of SEMA7A and ITGA6 high and negative fibroblasts was quantified (*n* = 4 per group). **(G)** Overexpression of SEMA7A was confirmed by Western blotting. **(H)** Cell-surface expression of SEMA7A in SEMA7A overexpression and control fibroblasts. **(I and J)** Representative images (I) and index (J; *n* = 3 per group) of migration and invasion of SEMA7A overexpression fibroblasts. **(K)** Quantification of percentage of SEMA7A^+^ fibroblasts by flow cytometry on freshly isolated normal and IPF human lungs (Normal, *n* = 4; IPF, *n* = 7). **(L and M)** Trichrome staining (L) and hydroxyproline (M; *n* = 10 per group) of mice lungs receiving SEMA7A high and negative fibroblasts, and age-matched mice were treated with culture medium only. Dash-boxed regions were shown at higher magnification. CTL, control; OE, overexpression. Scale bar: 1 mm (D and I), 500 μm (L). Three or four independent experiments were performed on fibroblasts from different patients (B, E, F, J, K, and M). Data are the mean ± SEM. *, P < 0.05; **, P < 0.01; ***, P < 0.001; ****, P < 0.0001 by Student’s *t* test (B, E, F, and I–K) and two-way ANOVA (M). Source data are available for this figure: SourceData F2.

To further explore the functional roles of SEMA7A, F3, and ITGA6 in fibroblast invasion, we flow sorted fibroblasts based on their cell-surface expression (Fig. 2 A) and validated their expression levels by qRT-PCR (Fig. 2 B) and Western blot (Fig. 2 C). We found that cell migration and invasion were significantly increased in the cell-surface protein high fibroblasts (Fig. 2, D and E), which suggested that these proteins could be used to mark invasive lung fibroblasts. Moreover, both SEMA7A^high^ and ITGA6^high^ fibroblasts showed higher adherence capacities compared to the negative controls (Fig. 2 F), consistent with previous reports (Geng et al., 2019).

**Figure S2.**
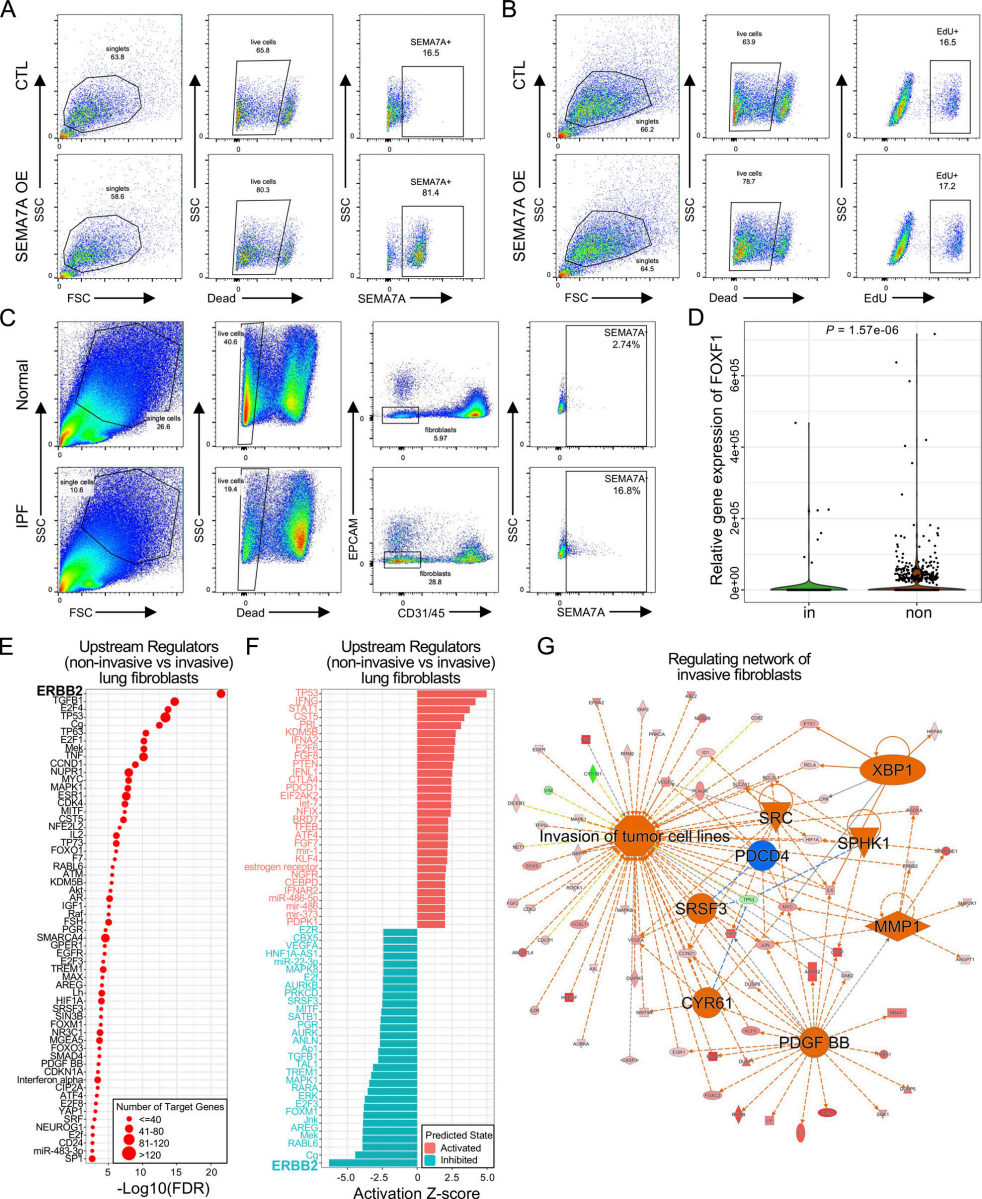
**ERBB2 (HER2) was the top inhibited upstream regulator in noninvasive fibroblasts. (A)** Flow cytometry analysis confirmed the overexpression of SEMA7A in lung fibroblasts. **(B)** Cell proliferation rates of fibroblasts with SEMA7A overexpression or control fibroblasts were determined by EdU assays. **(C)** Cell-surface expression of SEMA7A was determined by flow cytometry on single-cell homogenate of CD31^−^, CD45^−^, EPCAM^−^ cells from IPF and healthy samples. **(D)** Single-cell Western blot confirmed the downregulation of FOXF1 in invasive fibroblasts. **(E and F)** Dot plot visualization of the −Log_10_(FDR) (E) and bar plot visualization of the Activation Z-score (F) of the top 30 activated and inhibited upstream regulators of noninvasive fibroblasts by IPA analysis. ERBB2 was the top inhibited regulators of noninvasive fibroblasts. ERBB2 was highlighted as the most inhibited regulator. **(G)** The regulating network of invasive fibroblasts combining canonical signaling pathways and upstream regulators showed that the core signaling pathway was the invasion of tumor cell lines, suggesting that invasive lung fibroblasts had metastatic cancer-related signatures.

Functionally, overexpression of SEMA7A in lung fibroblasts (Fig. 2, G and H; and Fig. S2 A) dramatically increased the migration and invasion capacities of lung fibroblasts (Fig. 2, I and J) and slightly increased fibroblast proliferation (Fig. S2 B). Importantly, IPF lungs showed higher percentages of SEMA7A positive fibroblasts compared to normal lungs by flow cytometry analysis on freshly isolated lung fibroblasts (Fig. S2 C and Fig. 2 K) and this might explain the increased migration and invasion of IPF lung fibroblasts when compared to normal fibroblasts. As predicted, SEMA7A^high^ fibroblasts developed significantly more severe lung fibrosis than the mice receiving SEMA7A^negative^ fibroblasts and the mice without fibroblast injection in a humanized SCID IPF mouse model (NOD-scid-IL2Rγc^−/−^ [NSG] mice) in vivo (Geng et al., 2019; Trujillo et al., 2010), visualized by markedly increased pathologic lung remodeling, thickened alveolar walls and reduced alveolar area, Masson’s trichrome histologic staining (Fig. 2 L), and significantly elevated hydroxyproline content (Fig. 2 M). These data suggest that these cell-surface markers of invasive fibroblasts are not just “invasive markers,” but also functional in mediating fibroblast behaviors.

### Identification of transcription factors specific to invasive fibroblasts

**Figure 3.**
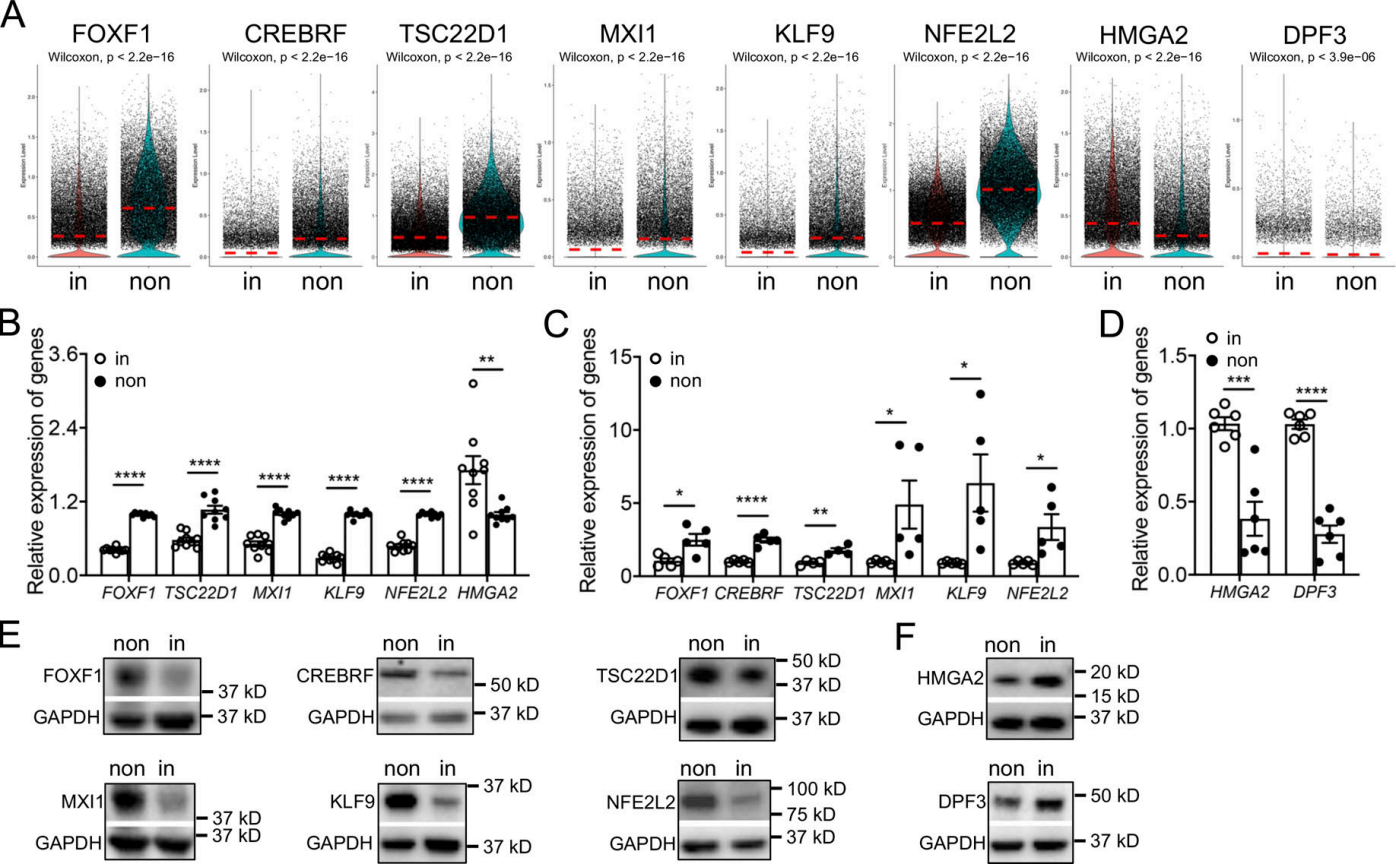
**Differentially expressed transcription factors in invasive and noninvasive fibroblasts. (A)** Visualization of differentially expressing transcription factors using violin plot in invasive and noninvasive fibroblasts. **(B–D)** Expression validation of transcription factors by bulk RNA-seq analysis (B) and qRT-PCR analysis (C and D). B, *n* = 9 per group; C, *n* = 4 for *TSC22D1* and *n* = 5 for other groups; D, *n* = 6 per group. **(E and F)** Western blot analysis of transcription factor expression in invasive and noninvasive fibroblasts. GAPDH served as loading control. non, noninvasive fibroblasts; in, invasive fibroblasts. Three independent experiments were performed on fibroblasts from different patients (B–D). Data are the mean ± SEM. *, P < 0.05; **, P < 0.01; ***, P < 0.001; ****, P < 0.0001 by Student’s *t* test (B–D). Source data are available for this figure: SourceData F3.

Next, we attempted to identify the critical transcription factors, which show specific expression patterns and might regulate fibroblast invasion. By scRNA-seq analysis, among the differentially expressed transcription factors, we found that mRNA levels of *FOXF1*, *CREBRF*, *TSC22D1*, *MXI1*, *KLF9*, and *NFE2L2* were significantly downregulated in invasive fibroblasts, while the expressions of *HMGA2* and *DPF3* were elevated in invasive fibroblasts (Fig. S1 G and Fig. 3 A). These data were validated by published bulk RNA-seq data (Fig. 3 B), qRT-PCR (Fig. 3, C and D), and Western blot (Fig. 3, E and F).

**Figure 4.**
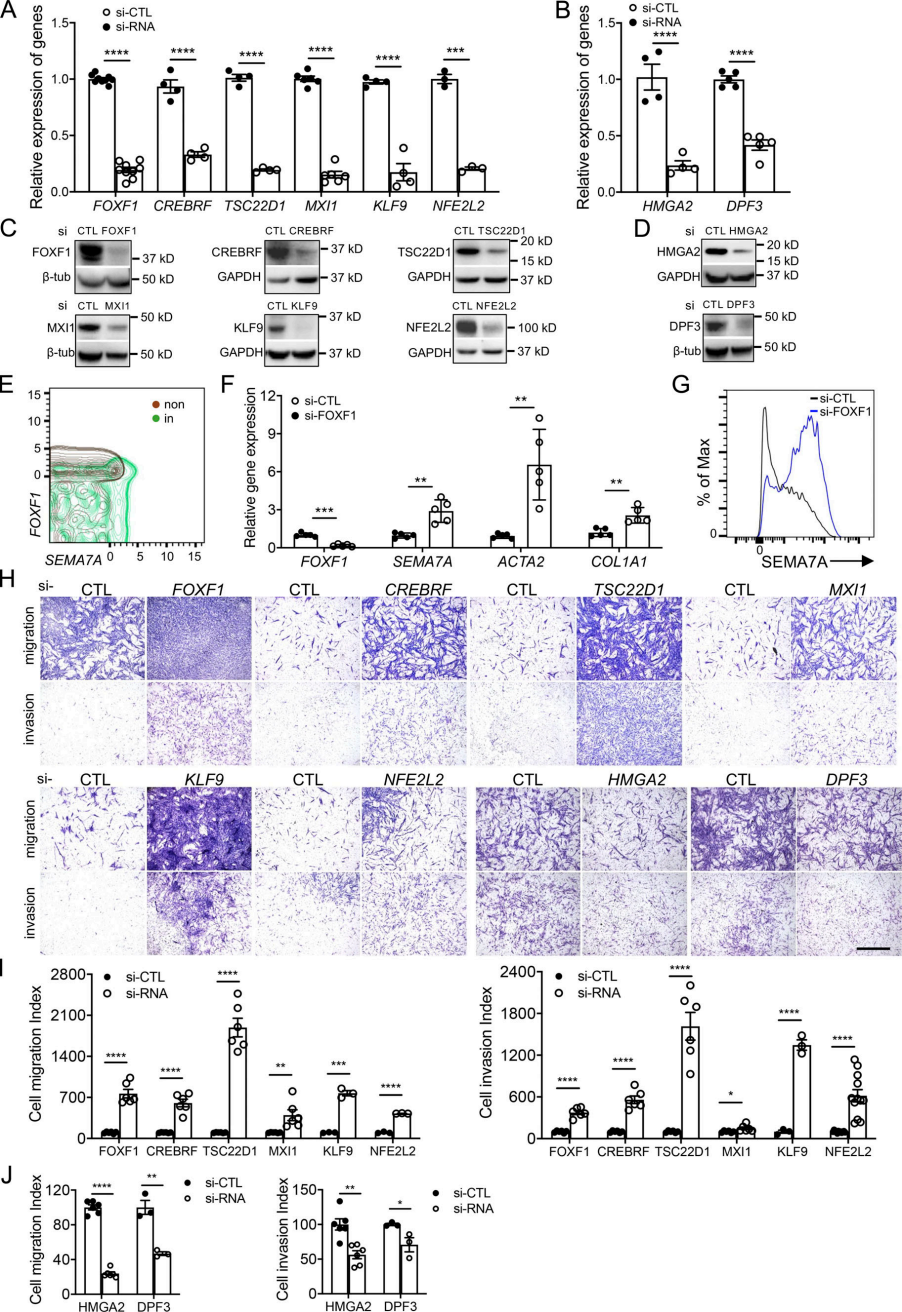
**Transcription factors regulated lung fibroblast invasion. (A–D)** Knockdown of transcriptional factors was confirmed by qRT-PCR (A and B) and Western blotting (C and D). A, *n* = 9 for *FOXF1*, *n* = 4 for *CREBRF*, *TSC22D1,* and *KLF9*, *n* = 6 for *MXI1*, *n* = 3 for *NFE2L2*; B, *n* = 4 for *HMGA2*, *n* = 5 for *DPF3*. **(E)** *FOXF1* and *SEMA7A* expressions showed negative correlation in scRNA-seq by SeqGeq. **(F and G)** Relative mRNA levels of *FOXF1*, *SEMA7A*, and collagen-related protein gene, *ACTA2* and *COL1A1* (F, *n* = 5 per group) and cell-surface expression of SEMA7A (G) after *FOXF1* knockdown. **(H–J)** Representative images (H) and index quantification (I and J) of migration and invasion of fibroblasts after knockdown assay. I, FOXF1, CREBRF, TSC22D1, and MXI1, *n* = 6 for migration and invasion, KLF9, *n* = 3 for migration and invasion, NFE2L2, *n* = 3 for migration and *n* = 11 for invasion; J, HMGA2, *n* = 6 for migration and invasion, DPF3, *n* = 3 for migration and invasion. CTL, control; non, noninvasive; in, invasive. Three or four independent experiments were performed on fibroblasts from different patients (A, B, F, I, and J). Data are the mean ± SEM. Scale bar: 1 mm. *, P < 0.05; **, P < 0.01; ***, P < 0.001; ****, P < 0.0001 by Student’s *t* test. Source data are available for this figure: SourceData F4.

To confirm that these transcription factors were necessary in mediating the invasiveness of lung fibroblasts, we performed siRNA knockdown assays for all the transcription factors identified. qRT-PCR (Fig. 4, A and B) and Western blot analysis (Fig. 4, C and D) were performed to evaluate the efficiency of the knockdown assays.

Foxf1 was recently reported to inhibit myofibroblast invasion, collagen secretion, and pulmonary fibrosis in a bleomycin-induced fibrosis mouse model (Black et al., 2018). Here in our scRNA-seq data, *FOXF1* was significantly downregulated in invasive fibroblasts, which was further confirmed by single-cell Western blot (Fig. S2 D), and showed negative correlation with the expression of *SEMA7A* (Fig. 4 E), which we defined as an invasive fibroblast surface marker. Knockdown of *FOXF1* in lung fibroblasts from IPF patients significantly increased the expression of fibrosis associated markers at mRNA levels including *ACTA2* and *COL1A1* (Fig. 4 F), as well as mRNA level (Fig. 4 F) and cell-surface protein level (Fig. 4 G) of SEMA7A.

Knockdown of *FOXF1*, *CREBRF*, *TSC22D1*, *MXI1*, *KLF9*, and *NFE2L2* significantly promoted fibroblast migration and invasion (Fig. 4, H and I), while *HMGA2* and *DPF3* deficiency blunted fibroblast migration and invasion (Fig. 4, H and J). These data suggested that these transcription factors drove a genetic program in mediating fibroblast migration and invasion in human lungs.

### Invasive fibroblasts shared similar regulatory programs with metastatic adenocarcinoma

**Figure 5.**
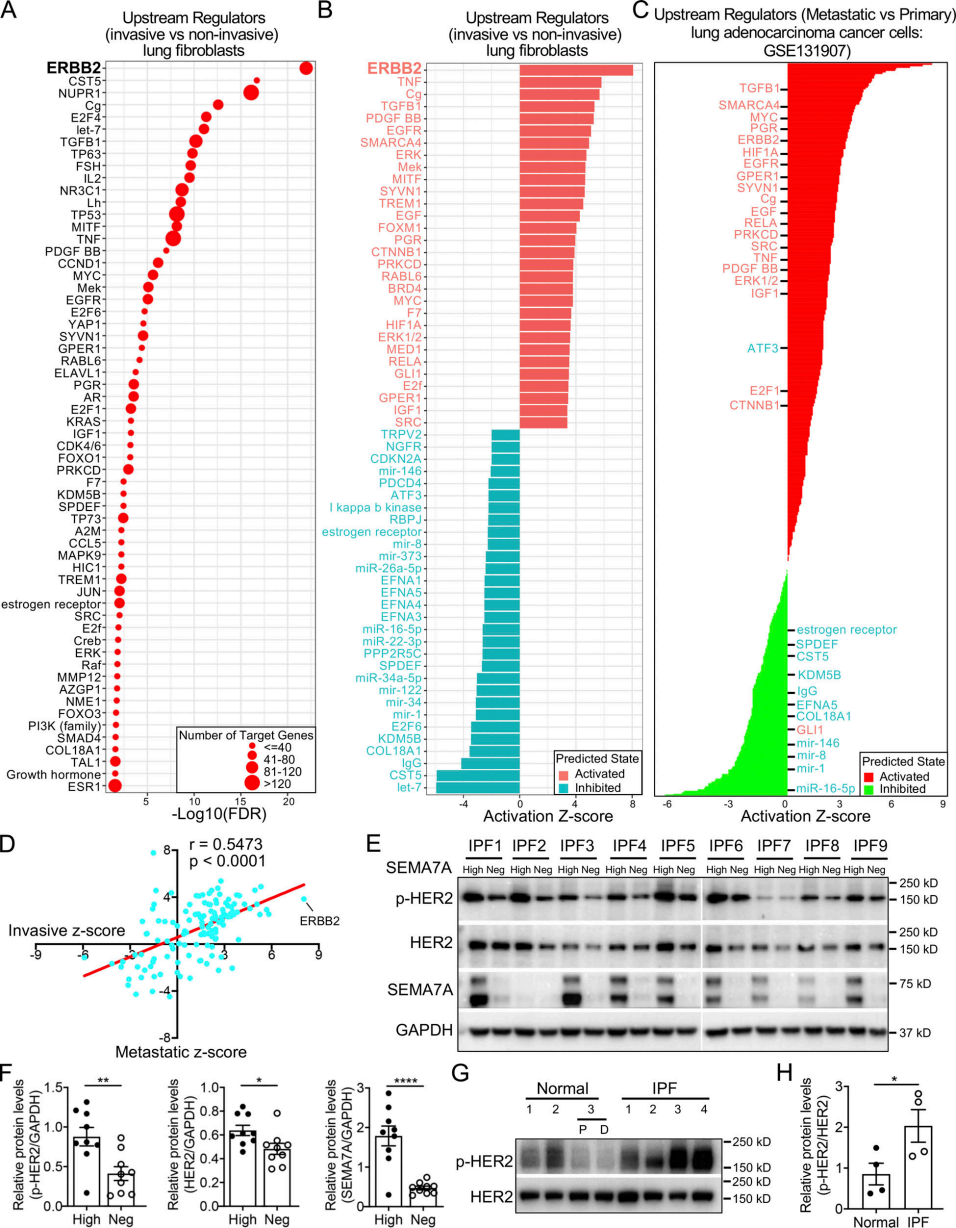
**Significantly activated ERBB2 (HER2) signaling pathway in invasive fibroblasts. (A and B)** Dot plot visualization of the −Log_10_(FDR) (A) and bar plot visualization of the activation Z-score (B) of the top 30 activated and inhibited upstream regulators of invasive fibroblasts by IPA analysis. ERBB2 was the most activated regulators of invasive fibroblasts. ERBB2 was highlighted as the most activated regulator. **(C)** IPA analysis revealed the upstream regulators of metastatic lung adenocarcinoma cancer cells compared to primary cancer cells retrieved from GSE131907. Most of the top activated/inhibited (listed in red/green texts, respectively) upstream regulators of invasive fibroblasts were these of metastatic cancer cells. **(D)** Pearson correlation analysis of activation z-score of shared upstream regulators (*n* = 129) of invasive fibroblasts versus metastatic lung adenocarcinoma cancer cell. Linear regression analysis was performed and visualized in red line. **(E)** p-HER2, total HER2, and SEMA7A protein levels in sorted SEMA7A high and negative fibroblasts in nine IPF fibroblast lines were determined by Western blot. GAPDH served as loading control. **(F)** Quantification of the Western blot was used to determine the relative protein levels of p-HER2, total HER2, and SEMA7A in E (*n* = 9 per group). **(G and H)** p-HER2 and total HER2 in sorted fibroblasts from three normal and four IPF lung were determined by Western blot and quantification was performed (H; *n* = 4). Neg, megative; P, proximal lung regions; D, distal lung regions. Two independent experiments were performed on fibroblasts from different patients (F and H). Data are the mean ± SEM. *, P < 0.05; **, P < 0.01; ****, P < 0.0001 by Student’s *t* test (F and H). Source data are available for this figure: SourceData F5.

To evaluate the signaling pathways and upstream regulators that were potentially mediating the invasive gene expression profile, the specific genes of invasive and noninvasive fibroblasts were imported into IPA, a tool to investigate possible interactions of differentially regulated signaling pathways. Interestingly, the ERBB2 (HER2) was the most activated upstream regulator in the invasive fibroblasts indicated by the P value of overlap (Fig. 5 A) and activation z-score (Fig. 5 B). In contrast, ERBB2 was the most inhibited upstream regulator in noninvasive fibroblasts also indicated by the P value of overlap (Fig. S2 E) and activation z-score (Fig. S2 F). These data suggest that the ERBB2 signaling pathway might be a key regulator driving lung fibroblast invasion.

**Figure S3.**
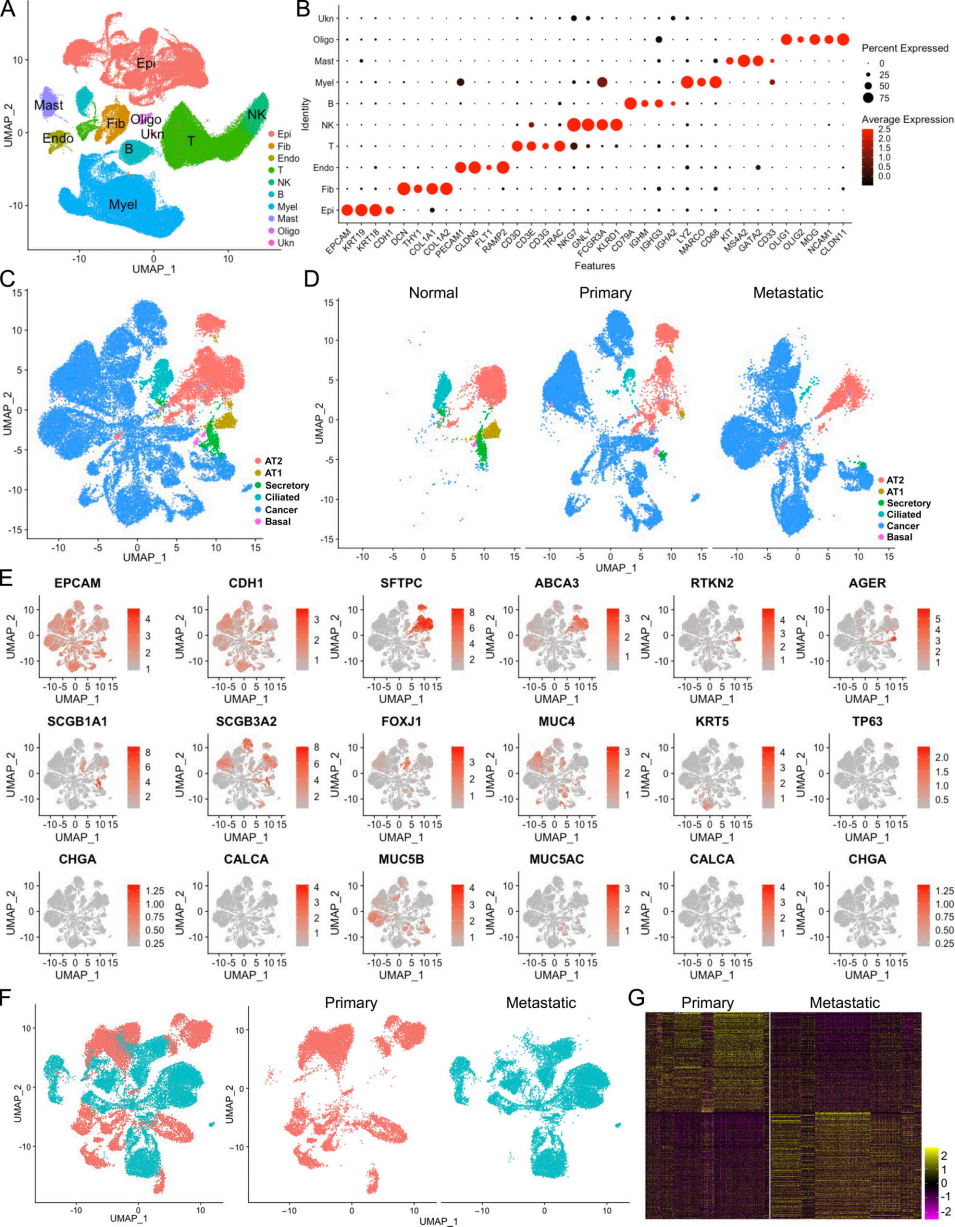
**Retrieval of scRNA-seq on primary and metastatic lung adenocarcinoma from** GSE131907**. (A and B)** Cell-type identification (A) by the expression of canonical cell-type marker gene expression (B). Epi, epithelial cell lineage; Fib, fibroblast; Endo, endothelial cell; Myel, myeloid cell; NK, natural killer; Oligo, Oligodendrocyte; Ukn, unknown cell. **(C–E)** Epithelial cell lineage extraction and definition (C) and distribution (D) of epithelial cell type by the expression of canonical epithelial cell-type marker genes (E). **(F)** Extraction and distribution of cancer cell from primary and metastatic tumors. **(G)** Heatmap of top 500 genes of primary and metastatic cancer cells.

Many of the top upstream regulators of invasive fibroblasts, including ERBB2 (Oh and Bang, 2020), EGFR (Robichaux et al., 2021), TNF (Wajant et al., 2003), TP63 (Melino, 2011), TP53 (Powell et al., 2014), and SMARCA4 (Concepcion et al., 2021), have been shown to be involved in tumor metastasis (Fig. 5, A and B). The regulatory network by IPA analysis combining the upstream regulators and the canonical pathways revealed tumor cell invasion signaling as the core-activated pathway of the network (Fig. S2 G). Based on these observations, we hypothesized that the regulatory programs of the invasive fibroblasts were closely associated with the signaling pathways promoting tumor metastasis. To confirm that, a recently published scRNA-seq dataset on normal lung tissue and primary and metastatic lung adenocarcinoma was reaccessed and the primary and metastatic cancer cells and their specific gene expression programs were identified (Fig. S3, A–G). IPA analysis revealed the upstream regulators of the metastatic cancer cells based on the specific gene expression (Fig. 5 C). Surprisingly, among the top activated upstream regulators of invasive fibroblasts, most were significantly activated in metastatic cancer cells (20/30), while many of the inhibited upstream regulators of invasive fibroblasts were also appreciably inhibited in metastatic cancer cells (11/30; Fig. 5 C). Analysis of all the shared upstream regulators showed significant positive correlations between invasive fibroblasts and metastatic cancer cells (Fig. 5 D). These data raised the hypothesis that invasive lung fibroblasts shared similar genetic regulatory programs with metastatic lung cancer cells which were possibly orchestrated by HER2 signaling.

### Activated ERBB2 (HER2) signaling in invasive lung fibroblasts

As described above, ERBB2 (HER2) was the most activated regulator in invasive fibroblasts and the top one inhibited regulator in noninvasive fibroblasts (Fig. 5, A and B; and Fig. S2, E and F). To confirm that, we sorted SEMA7A high and negative fibroblasts as invasive and noninvasive fibroblasts by FACS from fibroblasts of nine IPF patients. Western blot analysis showed that SEMA7A^high^ fibroblasts showed significantly elevated HER2 phosphorylation (p-HER2) and SEMA7A expression compared to SEMA7A^negative^ fibroblasts, and the total HER2 protein levels were also increased in most patients (Fig. 5, E and F).

**Figure S4.**
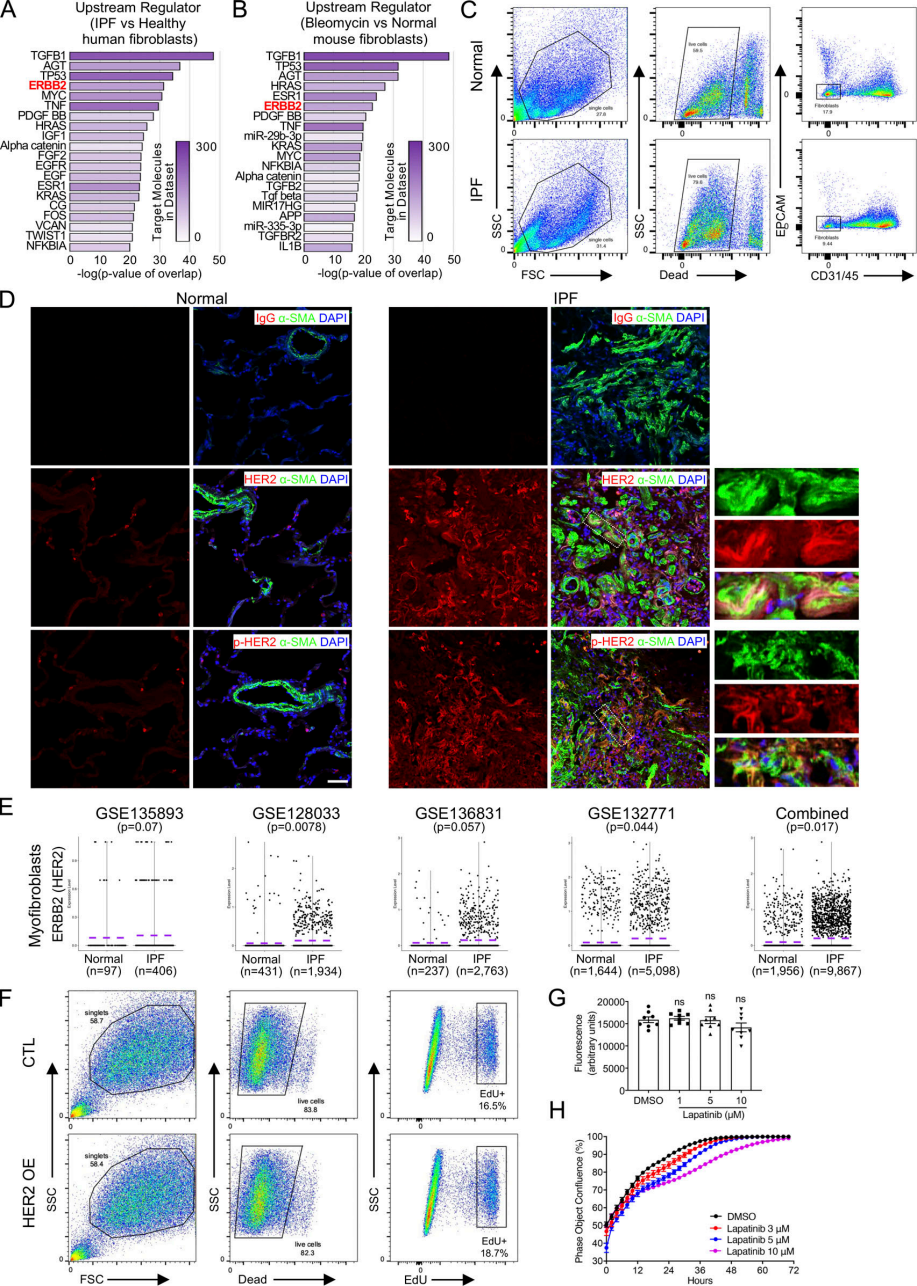
**ERBB2 was the top upstream regulator in fibrotic fibroblasts in human and mouse lungs. (A and B)** ERBB2 was the top upstream regulator of both human IPF lung fibroblasts (A) and bleomycin-induced mouse lung fibrotic fibroblasts (B). **(C)** Sorting strategy for normal and IPF lung fibroblasts for p-HER2 and total HER2 Western blot. **(D)** Immunostaining of HER2/p-HER2 with activated fibroblast (myofibroblast) marker, α-SMA, in normal and IPF human lung cryosections. IgG isotype was used as control for HER2 and p-HER2 staining. Higher magnifications of the boxed regions were provided. **(E)** Transcription of ERBB2 (HER2) in myofibroblasts in published scRNA-seq datasets. P value of each comparison, cell number (*n*) of each group, and average expressions of ERBB2 (purple dotted line) were included. **(F)** Cell proliferation rates of normal fibroblasts with HER2 overexpression or control fibroblasts were determined by EdU assays. **(G)** Fibroblast viability after Lapatinib treatment was determined by Calcein AM Assay (*n* = 8 per group). **(H)** Fibroblast growth rate after treatment of Lapatinib at increasing concentration (*n* = 6 per group). Three independent experiments were performed on fibroblasts from different patients (G and H). Data are the mean ± SEM. ns, not significant by two-way ANOVA (G). Scale bars, 20 μm (D).

To determine if HER2 signaling was also activated under in vivo fibrosis conditions, we examined the upstream regulators of fibrotic fibroblasts in our recently published analysis (Liu et al., 2021). Consistently, ERBB2 was one of the top activated upstream regulators in both human IPF lung fibroblasts (Fig. S4 A) and bleomycin induced mouse fibrotic lung fibroblasts (Fig. S4 B). To further support this, freshly sorted fibroblasts (Fig. S4 C) from normal and IPF human lungs were lysed for Western blot and significantly elevated p-HER2 levels were found in IPF lung fibroblasts compared to fibroblasts from normal lungs (Fig. 5, G and H). We observed costaining of the activated fibroblast (myofibroblast) marker, α-SMA within the fibrotic foci in the IPF lung sections, and activation (p-HER2) of HER2 in fibroblasts, although HER2/p-HER2 expression was also apparent in epithelial cells in normal lungs and in blood cells in IPF lungs (Fig. S4 D). The elevated HER2 transcription in myofibroblasts from IPF lungs was further confirmed by analyses of published scRNA-seq datasets (Fig. S4 E; Adams et al., 2020; Habermann et al., 2020; Liu et al., 2021; Morse et al., 2019; Tsukui et al., 2020). These data confirmed that the HER2 signaling was activated in SEMA7A^high^ invasive and IPF lung fibroblasts.

### HER2 activation promoted fibroblast invasion and fibrosis

**Figure 6.**
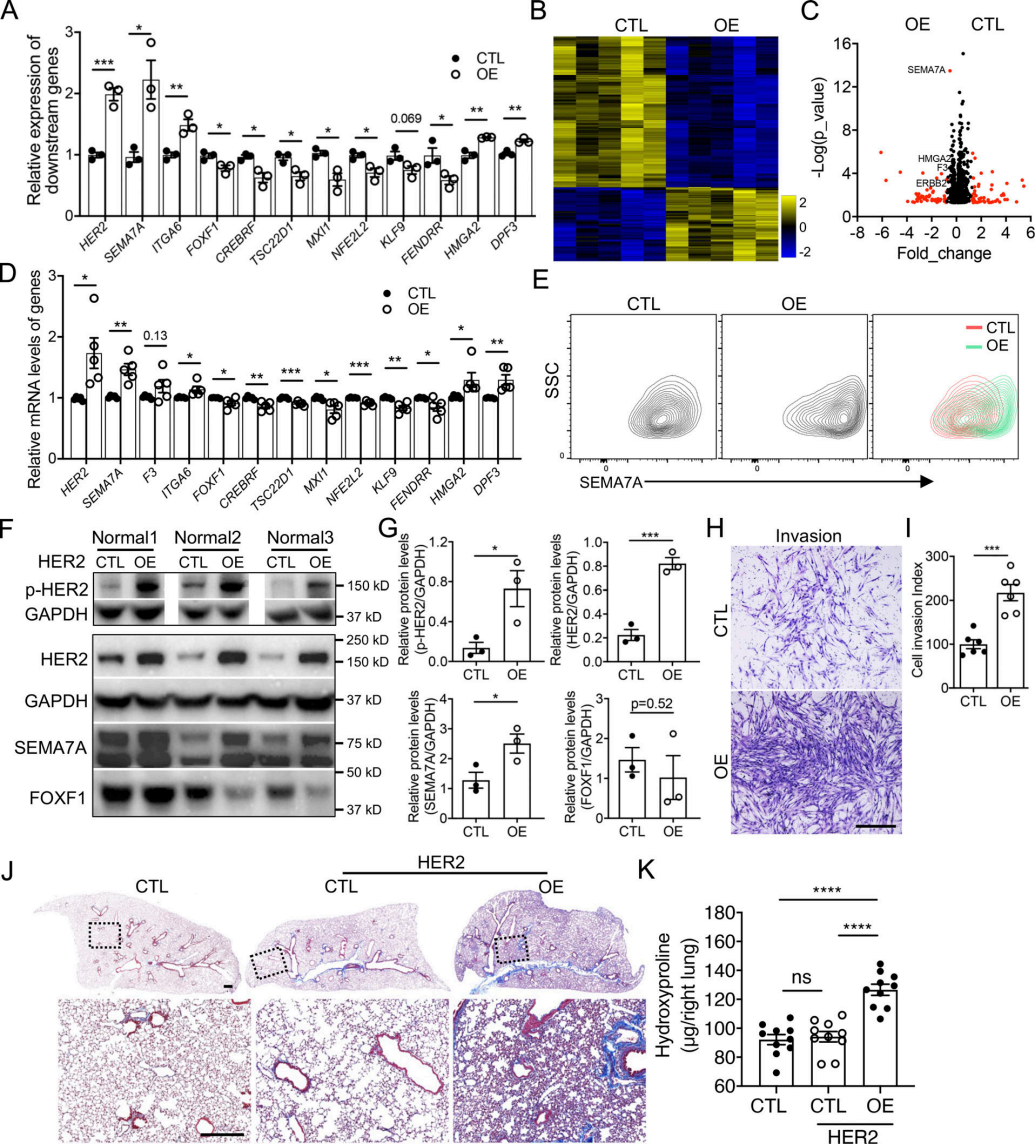
**HER2 signaling activation increased fibroblast invasion and fibrosis. (A)** The expression of invasive and noninvasive specific genes in HER2 overexpression normal fibroblasts were detected by qRT-PCR (*n* = 3 per group). **(B)** Heatmap of the differentially expressed genes of control and HER2 overexpressing normal human lung fibroblasts by bulk RNA-seq. **(C)** Volcano plot of the top differentially expressed genes between control and HER2 overexpressed normal human lung fibroblasts by bulk RNA-seq. Red dots indicated the genes at Fold_change >0.5 and black dots indicated the genes at Fold_change ≤0.5. **(D)** Relative expression of invasive and noninvasive specific genes in HER2 overexpression normal fibroblasts detected by bulk RNA-seq (*n* = 5 per group). **(E)** Upregulated cell-surface expression of SEMA7A in HER2 overexpression normal lung fibroblasts was confirmed by flow cytometry analysis. **(F and G)** Western blotting confirmation of the expression of p-HER2, HER2, SEMA7A, and FOXF1 in HER2 overexpression normal human lung fibroblasts (F) and quantification of the densitometry (G; *n* = 3 per group). GAPDH served as loading control. **(H and I)** Representative images (H) and index quantification (I; *n* = 6 per group) of normal lung fibroblast invasion after HER2 overexpression. **(J and K)** Trichrome staining (J) and hydroxyproline (K; *n* = 10 per group) of mice lungs receiving HER2 overexpressing and control normal human lung fibroblasts, and age-matched mice were treated with culture medium only. Dash-boxed regions were shown at higher magnification. CTL, control; OE, overexpression. Scale bar: 1 mm (H) and 500 μm (J). Three or four independent experiments were performed on fibroblasts from different patients (A, D, G, I, and K). Data are the mean ± SEM. *, P < 0.05; **, P < 0.01; ***, P < 0.001; ****, P < 0.0001 by Student’s *t* test (A, D, G, and I) and two-way ANOVA (K). Source data are available for this figure: SourceData F6.

Next, we attempted to determine if activation of HER2 is a causal factor for fibroblast invasion. To confirm this, we generated HER2 overexpressing stable lines in normal human lung fibroblasts by lentivirus infection. The overexpression efficiency and activation of HER2 were confirmed by increased total protein levels and phosphorylation levels of HER2 in these stable lines (Fig. 6, F and G). Interestingly, the mRNA levels of invasive specific genes were elevated, while those of noninvasive specific genes were downregulated by HER2 activation in normal lung fibroblasts, confirmed by both qRT-PCR (Fig. 6 A) and bulk RNA-seq (Fig. 6, B–D). The cell-surface protein levels (Fig. 6 E) and the total protein levels (Fig. 6, F and G) of SEMA7A, a representative invasive cell-surface marker confirmed above, were also significantly increased, while FOXF1, the transcriptional factor specific to noninvasive fibroblasts, was downregulated in HER2-activated fibroblasts (Fig. 6, F and G). These data support the hypothesis that HER2 activation activates genetic signatures to support fibroblast invasion and subsequently increase invasive capacity of normal lung fibroblasts. Interestingly, HER2 overexpression had limited effects on fibroblast proliferation (Fig. S4 F), whereas the in vitro invasion assay and in vivo SCID IPF mouse model showed an increased cell invasion index and abundant collagen deposition caused by the HER2 activated normal lung fibroblasts, demonstrated by a significant elevation in fibroblast invasion (Fig. 6, H and I) and a dramatic increase in lung fibrotic remodeling and hydroxyproline content (Fig. 6, J and K).

### HER2 deficiency rescued the invasion related gene signatures

**Figure 7.**
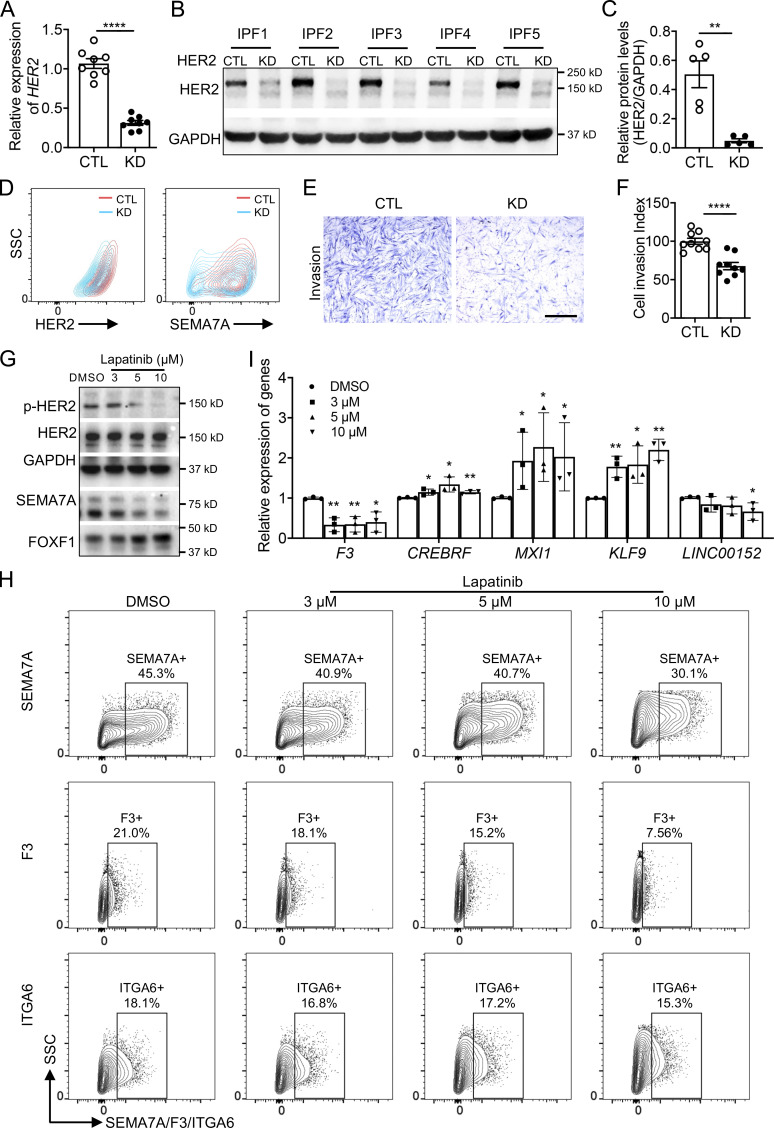
**HER2 deficiency rescued the dysregulated gene profiles in IPF lung fibroblasts. (A–C)** HER2 knockdown efficiency was confirmed by qRT-PCR (A) and Western blotting (B and C; A, *n* = 8 per group; C, *n* = 5 per group). **(D)** Cell-surface protein level of HER2 and SEMA7A in HER2 knockdown IPF lung fibroblasts. **(E and F)** Representative images (E) and index quantification (F; *n* = 9 per group) of fibroblast invasion after HER2 knockdown. **(G)** Protein levels of p-HER2, total HER2, SEMA7A, and FOXF1 in IPF lung fibroblasts after treatment of HER2 inhibitor, Lapatinib, at increasing concentrations. **(H)** Downregulation of cell-surface expression of SEMA7A, F3, and ITGA6 in Lapatinib-treated fibroblasts was determined by flow cytometry analysis. **(I)** Transcription levels of other representative genes in IPF lung fibroblasts after Lapatinib treatment were determined by qRT-PCR (*n* = 3 per group). Scale bar: 1 mm (E). CTL, control; KD, knockdown. Three or four independent experiments were performed on fibroblasts from different patients (A, C, F, and I). Data are the mean ± SEM. *, P < 0.05; **, P < 0.01; ****, P < 0.0001 by Student’s *t* test (A, C, and F) or one-way ANOVA (I). Source data are available for this figure: SourceData F7.

As HER2 appears to drive the gene programs of fibroblast invasion and mediated lung fibrosis, we then attempted to knockdown HER2 to examine the effects on IPF lung fibroblast effector functions. HER2 knockdown efficiency was confirmed by qRT-PCR (Fig. 7 A) and Western blot (Fig. 7, B and C). Consistently, cell-surface expressions of HER2 and SEMA7A were also significantly decreased (Fig. 7 D). Functionally, HER2 deficiency also caused a dramatic decrease in IPF fibroblast invasion (Fig. 7, E and F), confirming the crucial role of HER2 signaling in regulating fibroblast invasion.

As HER2 is a well-studied gene in cancer, several molecularly targeted therapies of HER2 positive cancers have recently become available, and Lapatinib is one of the most effective ones (Geyer et al., 2006). After treating IPF lung fibroblasts with Lapatinib, decreased p-HER2 levels were observed in a dose-dependent manner (Fig. 7 G), suggesting that Lapatinib was effective in blocking HER2 signaling in IPF lung fibroblasts. Total protein levels of SEMA7A were decreased while FOXF1 increased with higher Lapatinib concentrations (Fig. 7 G). We also examined the cell-surface expression of SEMA7A, F3, and ITGA6, cell-surface proteins specific to invasive fibroblasts, and found decreased protein levels after Lapatinib treatment (Fig. 7 H). The RNA levels of the other representative genes specific to invasive or noninvasive fibroblasts were also reversed to a noninvasive state by Lapatinib treatment (Fig. 7 I). These data revealed that blocking HER2 signaling by Lapatinib potentially reversed the gene programs of invasive fibroblasts and might blunt fibroblast invasion and potentially fibrosis.

### Targeting HER2 blunted fibroblast invasion and ameliorated pulmonary fibrosis

**Figure 8.**
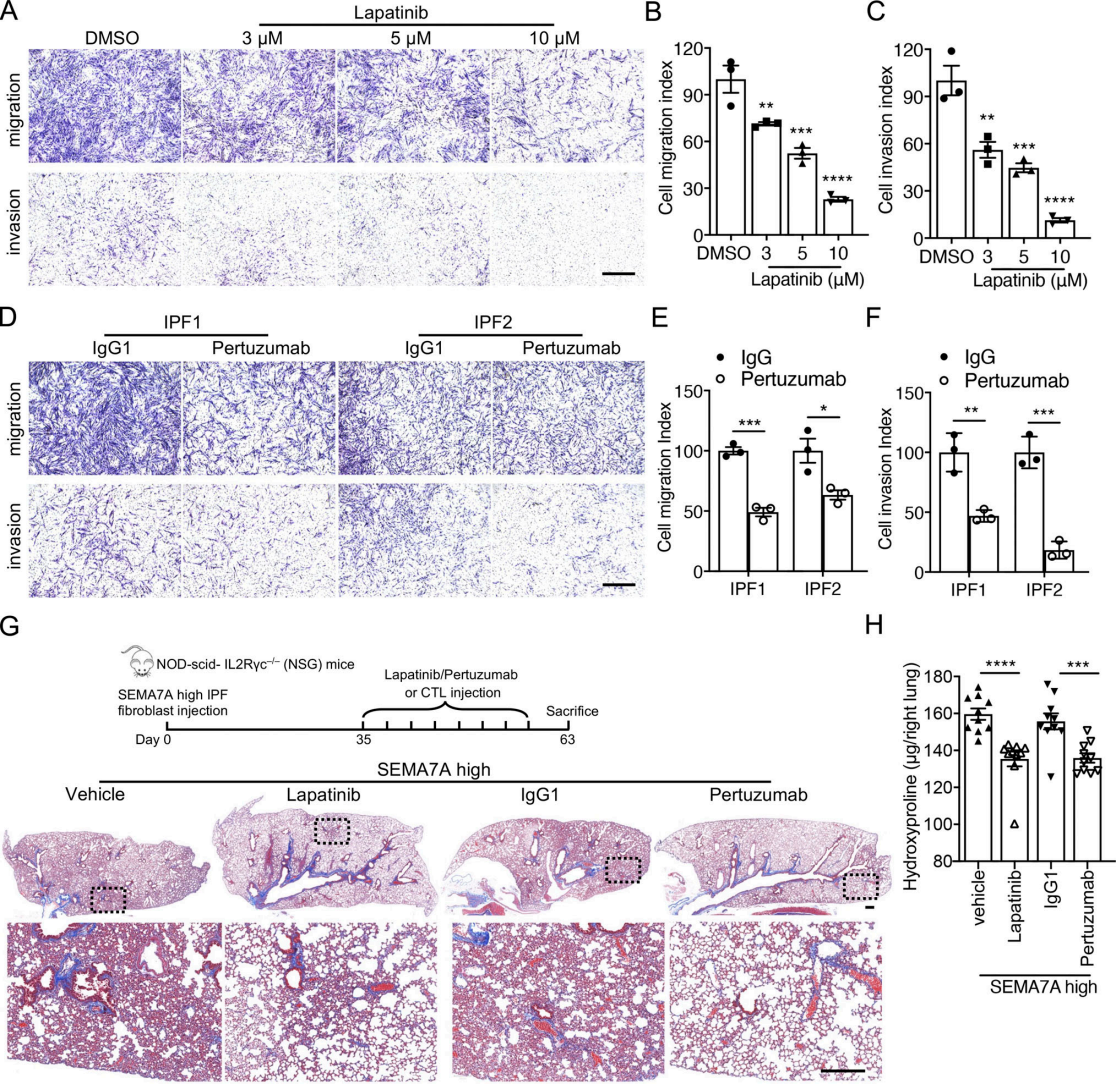
**Blocking HER2 signaling inhibited IPF lung fibroblast invasion and attenuated fibrosis. (A–C)** Representative images (A) and index quantification (B and C) of migration and invasion of fibroblasts treated with increasing doses of Lapatinib or DMSO (*n* = 3 per group). **(D–F)** Representative images (D) and index quantification (E and F) of migration and invasion of fibroblasts treated with Pertuzumab or IgG1 (*n* = 3 per group). **(G and H)** Masson’s trichrome staining of collagen in lung sections (G) and hydroxyproline content in lung tissues (H) from NSG mice injected with SEMA7A^high^ IPF fibroblasts and treated with Lapatinib, vehicle control, Pertuzumab, or IgG1 control (*n* = 10 per group). Dash-boxed regions were shown at higher magnification. Three independent experiments were performed on fibroblasts from different patients (B, C, E, and F). Data are the mean ± SEM. Scale bar: 1 mm (A and D) and 500 μm (G). *, P < 0.05; **, P < 0.01; ***, P < 0.001; and ****, P < 0.0001 by one-way ANOVA (B, C, E, and F) and two-way ANOVA (H).

**Figure S5.**
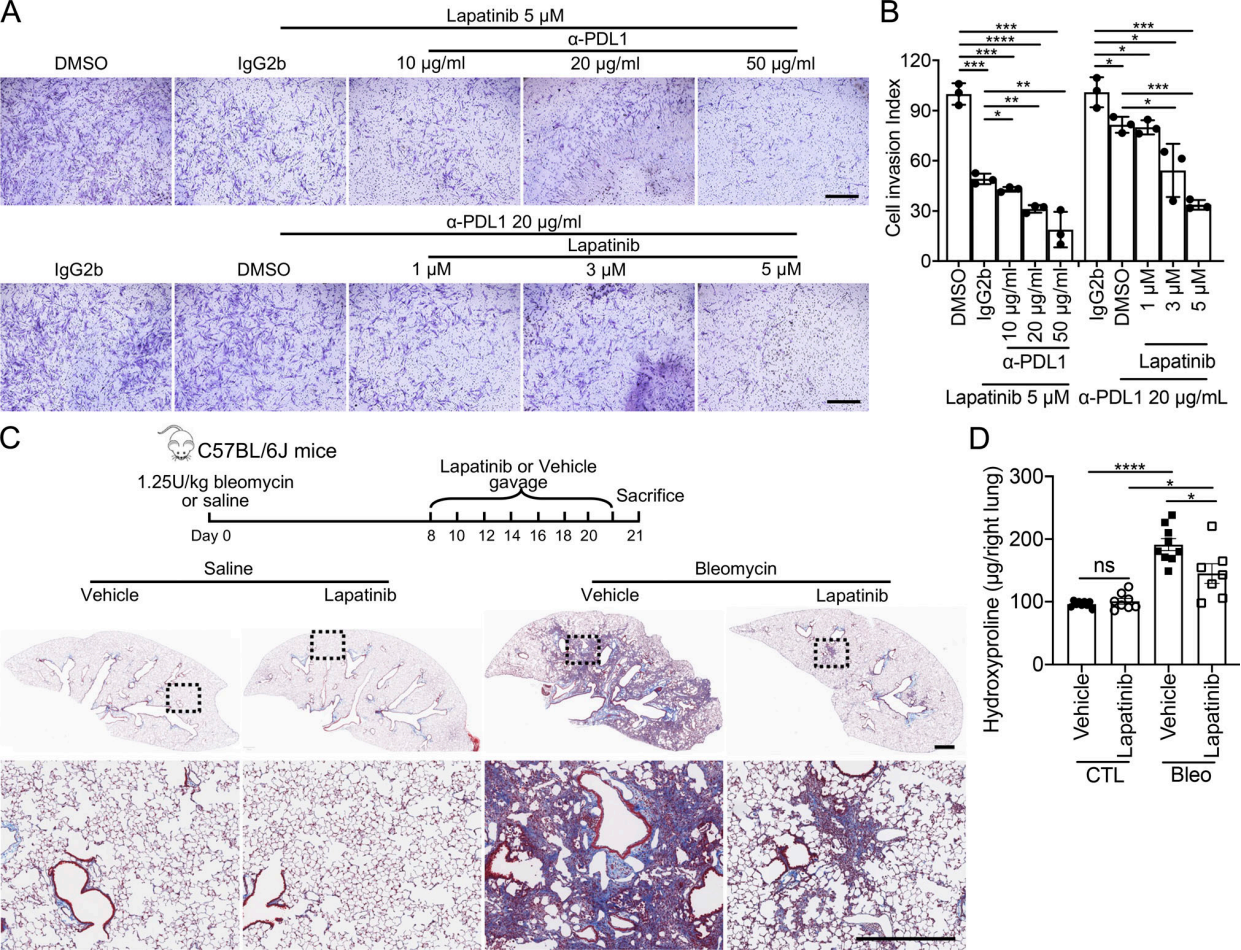
**Targeting HER2 blunted bleomycin-induced murine lung fibrosis. (A and B)** Fibroblast invasion (A) and invasion index (B) after combined treatment of Lapatinib and α-PDL1 (*n* = 3 per group). **(C and D)** Masson’s trichrome staining (C) and hydroxyproline content of lung tissues (D) from C57BL/6J mice injured with 1.25 U bleomycin and treated with Lapatinib or vehicle control. Dash-boxed regions were shown at higher magnification (control [CTL] with vehicle, *n* = 9; CTL with Lapatinib, *n* = 8; bleomycin with vehicle, *n* = 9; bleomycin with Lapatinib, *n* = 7). Three independent experiments were performed on fibroblasts from different patients (B and D). Data are the mean ± SEM. Scale bar: 1 mm (A) and 500 μm (C). *, P < 0.05; **, P < 0.01; ***, P < 0.001; and ****, P < 0.0001 by two-way ANOVA (B and D).

To further determine if blocking HER2 signaling had the potential to mediate IPF lung fibroblast invasion, we treated IPF lung fibroblasts with Lapatinib in migration and invasion assays. Similar to HER2 knockdown, Lapatinib inhibited fibroblast migration and invasion in a dose-dependent manner (Fig. 8, A–C) and showed little effect on fibroblast viability or growth (Fig. S4, G and H). We previously reported that PDL1 was elevated in invasive fibroblasts and anti-PDL1 (α-PDL1) antibody reduced fibroblast invasion although the mechanisms were less clear (Geng et al., 2019). We then combined Lapatinib and α-PDL1 in the invasion assay and found greater efficacy in inhibiting IPF fibroblast invasion (Fig. S5, A and B), suggesting there are nonredundant mechanisms. Treatment of IPF lung fibroblasts with the anti-HER2 monoclonal antibody, Pertuzumab (Perjeta), similarly reduced fibroblast migration and invasion (Fig. 8, D–F).

To confirm the effect of targeting HER2 in fibrosis, SEMA7A^high^ lung fibroblasts from IPF patients were sorted, enriched as invasive fibroblasts and injected into NSG mice. The mice were then treated with Lapatinib or Pertuzumab. Notably, mice treated with Pertuzumab or Lapatinib developed significantly less lung fibrosis compared to those treated with vehicle or IgG, visualized by reduced lung fibrotic remodeling, improved alveolar lung structures (Fig. 8 G), and significantly decreased hydroxyproline content in lung tissues (Fig. 8 H). These data suggested that blocking HER2 signaling ameliorated invasive fibroblast-induced lung fibrosis. Blocking HER2 in bleomycin induced mouse lung fibrosis model from day 7–20 after lung injury also remarkably reduced pulmonary fibrosis in C57bl/6 mice (Fig. S5, C and D).

**Figure 9.**
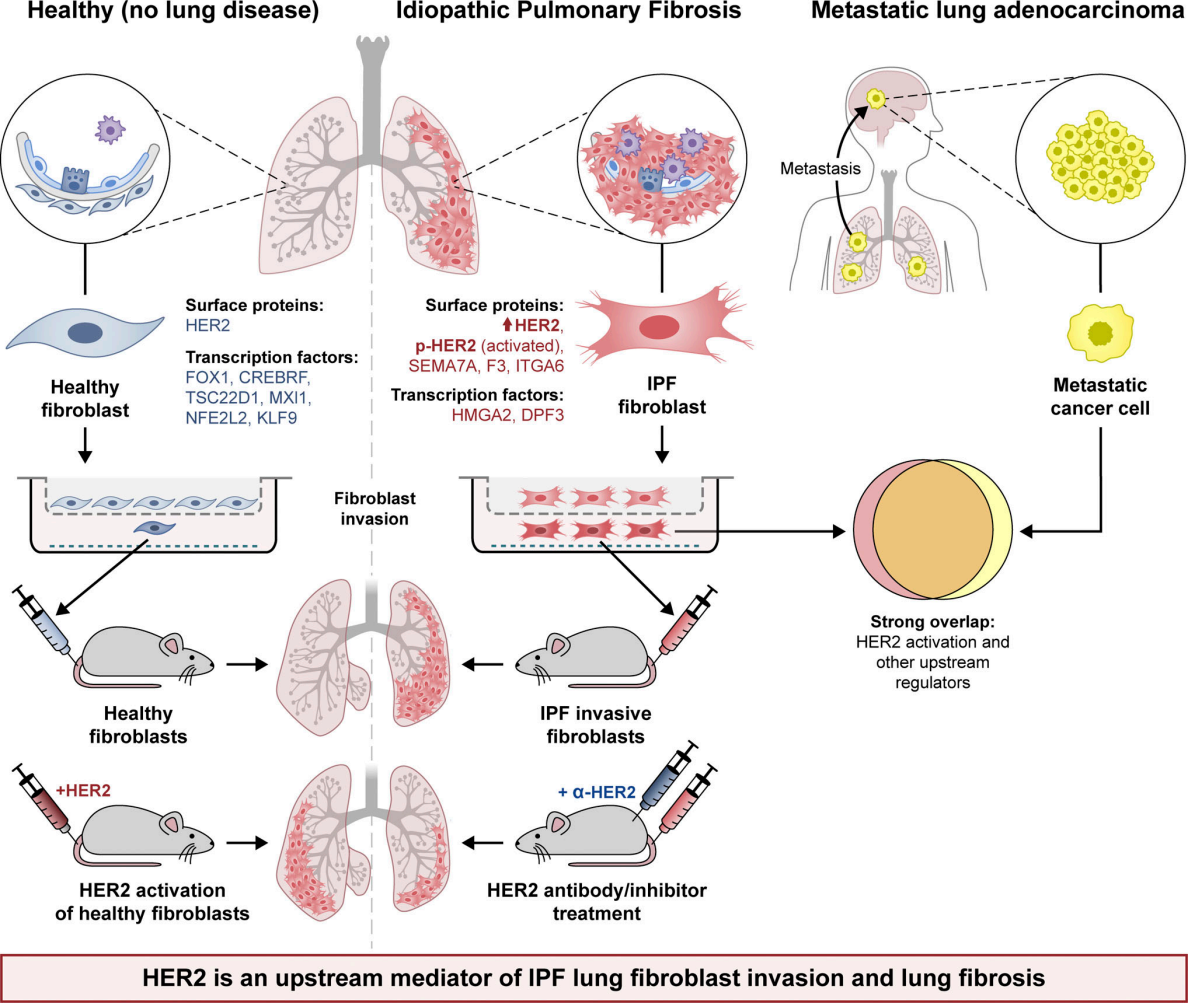
Schematic outline summarizing the genetic and regulatory programs of invasive IPF lung fibroblasts and the similarities to that of metastatic lung cancer cells, as well as their contribution to progressive lung fibrosis in a humanized mouse model for idiopathic pulmonary fibrosis.

In summary, through profiling of human lung fibroblasts derived from lung explants of IPF patients, we demonstrated gene expression programs of invasive and noninvasive fibroblasts. Within these differentially expressed genes, we further defined the functional roles of several cell-surface marker and transcription factor genes. More importantly, pathway analyses revealed a metastatic lung cancer–related regulatory program in invasive fibroblasts, and among these pathways HER2 was the most activated and significant one. Genetically or biochemically targeting HER2 had a dramatic effect in inhibiting lung fibroblast invasion and in rescuing IPF lung fibroblast-induced lung fibrosis (Fig. 9). All these data support the concept that that the HER2 signaling may be a key driver of lung fibroblast invasion in IPF and serve as an attractive target for therapeutic intervention of IPF.

## Discussion

IPF is a chronic, progressive, fibrotic interstitial lung disease of unknown cause and is the most common and lethal idiopathic interstitial pneumonia (King et al., 2014; Noble et al., 2012). Increasing evidence indicates that IPF is an epithelial-driven disease whereby a dysfunctional lung epithelium triggers the onset of fibroblast migration, proliferation, and differentiation (King et al., 2014; Noble et al., 2012). Activated fibroblasts secrete excessive amounts of ECM that subsequently remodel the lung architecture. Our previous studies as well as others’ have shown that a subset of fibrotic fibroblasts acquire an invasive phenotype that is essential for progressive fibrosis (Ahluwalia et al., 2016; Chen et al., 2016; Geng et al., 2019; Huan et al., 2015; Li et al., 2011; Lovgren et al., 2011; Xie et al., 2016). We recently discovered that the immune checkpoint ligand PDL1 (CD274) was upregulated on invasive fibroblasts and targeting PDL1 significantly inhibited fibroblast invasion in vitro and attenuated lung fibrosis in vivo (Geng et al., 2019). To gain additional insights into the molecular regulation of invasive fibroblasts, we profiled invasive and noninvasive fibroblasts by scRNA-seq and demonstrated a classification of invasive and noninvasive lung fibroblasts and their specific signature genes. Interestingly, combining all the differentially expressed genes of invasive fibroblasts for IPA analysis uncovered the cancer metastasis-related regulatory pathways in which the ERBB2 (HER2) signaling pathway was most activated in invasive fibroblasts. Blocking HER2 inhibited fibroblast migration and invasion and blunted lung fibrosis in a humanized SCID IPF model.

Several cell-surface markers were identified on invasive fibroblasts. SEMA7A (Semaphorin 7A, also called CD108) is a glycosylphosphatidylinositol-anchored Semaphorin that has been previously reported to be regulated by TGF-β1 and plays a critical role in TGF-β1–induced fibrotic responses (Kang et al., 2007). In the present study, SEMA7A was upregulated in invasive lung fibroblasts and SEMA7A^high^ fibroblasts induced more fibrogenesis than SEMA7A^negative^ fibroblasts. F3 (Coagulation Factor III, also called CD142 or tissue factor) is a cell membrane–associated protein that serves as the receptor and the essential cofactor for factors VII and VIIa (Mackman, 2004). F3 is dramatically increased in lungs from patients with IPF (Imokawa et al., 1997). In bleomycin-induced pulmonary fibrosis, the expression of F3 by alveolar macrophages, epithelial cells, and fibroblasts is dramatically increased (Olman et al., 1995). α6 (ITGA6, also called CD49f) containing integrins serve as cellular receptors for members of laminin family, a major structural component of the basement membrane. A recent report reveals that human IPF lung myofibroblasts express high levels of ITGA6 in vitro and in vivo and genetic ablation of ITGA6 in collagen-expressing mesenchymal cells protects mice against bleomycin injury-induced experimental lung fibrosis (Chen et al., 2016). These reports are consistent with our data.

Transcription factors are critical for cell transition. Based on scRNA-seq data, we identified several transcription factors that showed specific expression in invasive or noninvasive fibroblasts and that most of which have not yet been fully delineated in lung fibrosis. FOXF1, a member of the forkhead box family of transcription factors, has previously been shown to be critical for lung development (Mahlapuu et al., 2001), lung regeneration after partial pneumonectomy (Bolte et al., 2017), and the inhibition of bleomycin-induced pulmonary fibrosis (Black et al., 2018). TSC22D1 (TSC22 domain family, member 1) and MXI1 (MAX interactor 1) are also associated with pulmonary fibrosis in some transcriptomic, miRNomic (Granata et al., 2018), and methylation array data (Huang et al., 2014). NFE2L2 (NF-E2–related transcription factor 2, also called NRF2) stimulation increases the expression of KLF9 (Kruppel-like factor 9), resulting in increases in ROS production and subsequent cell death, however they show diverse functions in bleomycin-induced pulmonary fibrosis in mice (Cho et al., 2004; Hecker et al., 2014; Zucker et al., 2014) and IPF (Artaud-Macari et al., 2013). HMGA2 (High-mobility group AT-hook 2) is a transcription factor that is induced by the TGF-β1/Smad3 signaling pathway and is also reported to be upregulated in pulmonary fibrosis (Pandit et al., 2010; Song et al., 2013) and inhibit bleomycin-induced pulmonary fibrosis (Wang et al., 2016). We suggest that all the above transcription factors are parts of a signaling network to regulate fibroblast invasion and are potential factors for invasive to noninvasive fibroblasts transition and are potential therapeutic targets in IPF.

IPF has been reported to be associated with increased risk of lung cancer due to the occurrence of atypical or dysplastic epithelial changes in fibrosis which progressed to invasive malignancy (Park et al., 2001). In clinical studies, lung cancer is found either simultaneously in patients with IPF or during the follow-up of IPF patients and patients with IPF are nearly at five times more at risk to develop lung cancer than that of general population (Tzouvelekis et al., 2018). Despite abundant epidemiological and mechanistic links between IPF and lung cancer, little is known about the diagnostic and therapeutic management of these patients (Vancheri, 2013). In our previous study, we reported that tumor suppressor p53 negatively regulates PDL1 expression and fibroblast invasion (Geng et al., 2019). It is well known that loss of p53 promotes tumor metastasis (Powell et al., 2014). Here in this current study, by pathway analysis, we revealed high similarities between the regulatory programs of invasive IPF lung fibroblasts and metastatic lung adenocarcinoma cancer cells and the most significant one was HER2 signaling. Although commonly acceptable initiation processes and mechanisms of these two diseases differ significantly, many of the available lung cancer drugs are also effective in the treatment of fibrosis in many studies (Paliogiannis et al., 2021; Richeldi et al., 2014). Though more evidence should be provided to support the concept, the current study sheds light on the research value of cancer targets on pulmonary fibrosis.

HER2 (ERBB2) has been actively studied in cancer treatment for decades and was a breakthrough therapy for breast cancer. Anti-HER2 classes of drugs, such as trastuzumab, pertuzumab, lapatinib, and T-DM1, for HER2-positive breast cancer have been used to treat cancer (Pondé et al., 2018; Stern, 2012). Very limited reports have linked HER2 to pulmonary fibrosis. Targeting HER2 using 2C4, a monoclonal antibody directed against HER2 that blocks HER2/HER3 signaling, attenuated bleomycin-induced pulmonary fibrosis in mice (Faress et al., 1985), although the potential mechanisms remained undiscovered. Several reports linked EGFRs to pulmonary fibrosis by their expression levels and related genetic variation (Baughman et al., 1999; Epstein Shochet et al., 2019; Martinelli et al., 2011). However, the cellular and molecular mechanisms of EGFRs in pulmonary fibrosis in vivo or in vitro are lacking. In the present study, we combined the differentially expressed genes identified from invasive and noninvasive fibroblasts for IPA analysis and found the HER2 signaling is dramatically activated in invasive fibroblasts. Blocking the HER2 signaling with pertuzumab markedly inhibited lung fibrosis in a humanized mouse fibrosis model, suggesting that the HER2 signaling may be a key driver of fibroblast invasion and progressive lung fibrosis and may be a novel therapeutic target in IPF.

## Materials and methods

### Study approval

All human lung experiments were approved by the Cedars-Sinai Medical Center Institutional Review Board (IRB) and were in accordance with the guidelines outlined by the IRB. Informed consent was obtained from each subject (IRB: Pro00032727). All animal experiments were approved by the Institutional Animal Care and Use Committee at Cedars-Sinai Medical Center (protocol IACUC005136). All mice were housed in a pathogen-free facility at Cedars-Sinai Medical Center and had access to autoclaved water and pelleted mouse diet ad libitum.

### Human lung fibroblast culture and cell migration and invasion assays

Human lung fibroblasts were isolated and cultured as previously described (Geng et al., 2019). Briefly, human tissues from lung explants from patients underwent lung transplantation as well as normal donors were minced, digested, and cultured in DMEM supplemented with 15% FBS and antibiotic-antimycotic. The fibroblasts were maintained and expanded to passage 4 until the cells were pure and steadily growing and proliferating with a standard fibroblast morphology. Fibroblasts were used for in vitro and in vivo experiments at passage 4–8. Fibroblast migration and invasion assays were performed as previously described (Geng et al., 2019). In brief, fibroblasts were loaded into the top chamber of cell culture insert with 8.0 μm pore (for migration) or BioCoat Matrigel Invasion Chamber (for invasion). PDGF-BB (10 ng/ml, Peprotech) was used as a chemoattractant in the bottom chamber. After 24 h culture, cells passed through the matrigel layer and clung to the bottom of the insert membrane were determined as invasive fibroblasts. Cells remaining on the upper chamber were defined as noninvasive fibroblasts. For quantification of the migration and invasion index, the cells were fixed and stained with the Protocol Hema 3 stain set (Thermo Fisher Scientific) and counted in five randomly chosen fields per filter from triplicate filters per sample at ×40 magnification. For the cell collection for 10× Genomics scRNA-seq, the fibroblasts remaining on the upper chamber were collected by a scraper and trypsinized to make single-cell suspension of noninvasive fibroblasts, and the fibroblast clung to the bottom of the insert membrane were trypsinized to make single-cell suspension of invasive fibroblasts. These cells were used for 10× Genomics scRNA-seq analysis immediately without any further culture or recovery.

### scRNA-seq, data processing, and IPA

Sequencing library construction was done by the 10× Genomics chromium platform as previously described (Xie et al., 2018). Cell Ranger version 1.3.1 (10× Genomics) was used to process raw sequencing data and Cell Ranger R kit version 4.1.0 and Seurat suite version 4.1.0 for downstream analysis. Differentially expressed genes were extracted from invasive fibroblasts and noninvasive fibroblasts or primary and metastatic lung adenocarcinoma cancer cells for IPA using the cutoff: average expression >0.1, adjusted P value (false discovery rate [FDR]) <0.05, and absolute value of Log_2_ fold-change >0.58.

### Gene knockdown assay and qRT-PCT

siRNA knockdown assays were performed using Lipofectamin RNAiMAX Transfection Reagent following the manufacturer’s protocol (Thermo Fisher Scientific). Commercial siRNA used were listed: si-*FOXF1* (sc-60655; Santa Cruz Biotechnology), si-*CREBRF* (sc-91839; Santa Cruz Biotechnology), si-*TSC22D1* (16708; Thermo Fisher Scientific), si-*MXI1* (sc-35835; Santa Cruz Biotechnology), si-*KLF9* (sc-37716; Santa Cruz Biotechnology), si-*NFE2L2* (sc-37030; Santa Cruz Biotechnology), si-*HMGA2* (sc-37994; Santa Cruz Biotechnology) si-*DPF3* (sc-92150; Santa Cruz Biotechnology). Knockdown assay of *ERBB2* (HER2) was performed with lentivirus from (GeneCopoeia, LPPHCP267177L03-3-100) and HER2 overexpression lentivirus was customized from VectorBuilder. RNA was isolated using RNeasy Mini kit (Qiagen) following the manufacturer’s protocol. M-MLV Reverse Transcriptase (Promega) was used for cDNA synthesis. Gene expression was measured relative to the endogenous reference gene GAPDH using the comparative ΔCT method. The primer sequences used were listed in Table S2.

### Flow cytometry and FACS

Cells were resuspended in Hank’s balanced saline solution supplemented with 2% FBS, 10 mM Hepes, 0.1 mM EDTA, and antibiotic-antimycotic. Directly conjugated antibodies used were anti-SEMA7A-PE and anti-SEMA7A-BV480 (clone KS-2; BD Biosciences), anti-CD274-PE (clone 29E.2A3; Biolegend), anti-F3-PE (clone HTF-1; BD Biosciences), anti-ITGA6-PE (clone GoH3; BD Biosciences), APC anti-CD340 (erbB2/HER2, Clone 24D2; Biosciences), FITC anti-human CD326 (EpCAM, Clone 9C4; Biosciences), PE/Cy7 anti-human CD31 (Clone WM59; Biosciences), and PE/Cy7 anti-human CD45 (Clone HI30; Biosciences). DAPI was used to discriminate dead cells. Flow cytometry was performed using an LSRFortessa cell analyzer and FACS was performed on FACSAria III sorter (BD Immunocytometry Systems) and data were analyzed using FlowJo 10.2 software (Tree Star).

### Western blot

Western blot was performed as previously descripted (Geng et al., 2019). Antibodies were used: anti-GAPDH (clone 14C10; Cell Signaling Technology), anti-SEMA7A (NBP1-86555; Novus Biologicals), anti-F3 (clone E9M6T; Cell Signaling Technology), anti-ITGA6 (3750S; Cell Signaling Technology), anti-FOXF1 (AF4798; R&D system), anti-CREBRF (ab26262; Abcam), anti-TSC22D1 (NBP2-46238; Novus Biologicals), anti-MXI1 (A12098; ABclonal), anti-KLF9 (clone A-5; Santa Cruz Biotechnology), anti-NFE2L2 (clone D1Z9C; Cell Signaling Technology), anti-HMGA2 (8179s, clone D1A7; Cell Signaling Technology), anti-DPF3 (NBP2-14910; Novus Biologicals), anti-p-HER2 (6942s, clone D66B7; Cell Signaling Technology), and anti-HER2 (2165s, clone 29D8; Cell Signaling Technology). Secondary antibodies were anti-rabbit IgG, HRP-linked Antibody (7074s; Cell Signaling Technology), anti-mouse IgG, HRP-linked antibody (7076s; Cell Signaling Technology), and Peroxidase Donkey Anti-Goat IgG (H + L; 705-036-147; Jackson ImmunoResearch).

### Densitometry analysis

Densitometry quantitation of Western blots was performed with Adobe Photoshop (Luhtala and Parker, 2009). Briefly, the Western bands and adjacent background regions were chosen and selected at the same size by Rectangular Marquee Tool and the integrated densities of the bands were determined with background adjusted by Measurement Log and Recording Measurements. The relative protein levels were calculated by normalizing integrated density of the target proteins to that of loading control proteins.

### Immunofluorescence

Immunofluorescence was performed as previously described (Liu et al., 2021). In brief, freshly dissected tissues were fixed in 4% paraformaldehyde solutions (Thermo Fisher Scientific) in PBS overnight and the following day embedded in Optimal Cutting Temperature Compound and flash frozen. 10-μm cryosections were cut using a cryostat onto Superfrost Plus Microscope Slides. Immunofluorescence was performed using following primary antibodies overnight at 4°C: p-HER2 (6942s, clone D66B7; Cell Signaling Technology) and anti-HER2 (2165s, clone 29D8; Cell Signaling Technology), and Cy3-conjugated secondary antibody and FITC-conjugated α-SMA antibody (ab8211, clone 1A4; Abcam) were used to visualize the staining.

### Cell growth rate and cell viability assay

Cell growth rate of fibroblasts was measured by IncuCyte ZOOM Live Cell Analysis System (Essen BioScience). The viability of lapatinib-treated fibroblasts or control fibroblasts was examined by Calcein AM Cell Viability Assay Kit (4892-010-K; R&D systems).

### Humanized SCID mouse model of IPF

Humanized SCID mouse model of IPF, generated as previously reported (Trujillo et al., 2010), is a well-established humanized lung fibrosis mouse model, in which lung fibrosis is only observed after the injection of IPF but not normal lung fibroblasts. The lungs in this model show several pathological characteristics that are commonly diagnosed in human lungs of IPF patients, including nonresolving fibrotic lung remodeling, significantly increased matrix deposition, elevated profibrotic cytokine and chemokine secretion, and alveolar epithelial cell injury. Female NSG mice (6–8 wk old) were purchased from The Jackson Laboratory. The NSG mice received single-cell suspensions of SEMA7A high and negative IPF lung fibroblasts, or HER2 overexpression and control fibroblasts (0.5 × 10^6^ cells/mouse) via tail vein injection. For ERBB2 inhibitor studies, mice were treated with 0.5% CMS-Na (control group) or 50 mg/kg Lapatinib (Tocris Bioscience) every other day from day 35 to 63. For the anti-ERBB2 antibody studies, mice were injected with IgG1 (InVivoPlus human IgG1 isotype control, Bio X Cell) or anti-HER2 (Pertuzumab, kindly provided by Genentech) twice per week, 100 μg/mouse from day 35 to 63. Lung fibrosis was assessed on day 63 after fibroblast transfer. The left lobe was used for histology and right lobes were used for hydroxyproline assay. The assessment of lung fibrosis in this model could be judged by visualizing the lung remodeling histologically and pathologically, thickened alveolar wall and reduced alveolar area, Masson’s trichrome histologic staining in lung slides, and quantitatively hydroxyproline content in lung tissues (Habiel et al., 2018; Pierce et al., 2007).

### Bleomycin instillation

Bleomycin instillation has been previously described (Liu et al., 2021). Briefly, under anesthesia, mouse trachea was surgically exposed. Bleomycin (Hospira) in 25 μl PBS was instilled into the mouse trachea at a dose of 1.25 U/kg body weight. Control animals received same amount of saline alone. The tracheostomy site was sutured, and the animals were allowed to recover. For anti-ERBB2 treatment, mice received 0.5% CMS-Na (vehicle group) or 50 mg/kg Lapatinib (Tocris Bioscience) by gavage every other day from day 7 to 20. The mice were sacrificed on day 21. The left lobe was used for histology and right lobes were used for hydroxyproline assay.

### Statistical analysis

Data are expressed as the mean ± SEM. All experiments were repeated two or more times. Student’s two-tailed *t* test was used for comparing differences between two groups. One-way or two-way ANOVA followed by Tukey-Kramer test was used for multiple comparisons. Significance was set at P < 0.05. GraphPad Prism software 8.0 was used for statistical analysis.

### Online supplemental material

Fig. S1 shows identification of novel marker genes of invasive and noninvasive lung fibroblasts. Fig. S2 shows ERBB2 (HER2) was the top inhibited upstream regulator in noninvasive fibroblasts. Fig. S3 shows the retrieval of scRNA-seq on primary and metastatic lung adenocarcinoma from GSE131907. Fig. S4 shows HER2 (ERBB2) was the top upstream regulator of fibrotic fibroblasts in human and mouse lungs. Fig. S5 shows the targeting HER2 signaling blunted bleomycin-induced lung fibrosis. Table S1 shows human sample donor information and summary of scRNA-seq experiments. Table S2 shows the primer sequences for qRT-PCR.

## Data and materials availability

All data associated with this study are present in the paper or the supplementary figures and tables. scRNA-seq and total RNA-seq data have been uploaded to GEO (GSE137025 and GSE137026). Total RNA-seq on invasive and noninvasive IPF fibroblasts were published previously (accession number GSE118933). scRNA-seq data on healthy human lungs and primary and metastatic lung adenocarcinoma were retrieved from GSE131907.

## Supplementary Material

Table S1 shows human sample donor information and summary of scRNA-seq experiments.

Table S2 lists primer sequences for qRT-PCR.

SourceData F1 contains original blots for Fig. 1.

SourceData F2 contains original blots for Fig. 2.

SourceData F3 contains original blots for Fig. 3.

SourceData F4 contains original blots for Fig. 4.

SourceData F5 contains original blots for Fig. 5.

SourceData F6 contains original blots for Fig. 6.

SourceData F7 contains original blots for Fig. 7.

## References

[bib1] Adams, T.S., J.C. Schupp, S. Poli, E.A. Ayaub, N. Neumark, F. Ahangari, S.G. Chu, B.A. Raby, G. DeIuliis, M. Januszyk, . 2020. Single-cell RNA-seq reveals ectopic and aberrant lung-resident cell populations in idiopathic pulmonary fibrosis. Sci. Adv. 6:eaba1983. 10.1126/sciadv.aba1983 32832599 PMC7439502

[bib2] Ahluwalia, N., P.E. Grasberger, B.M. Mugo, C. Feghali-Bostwick, A. Pardo, M. Selman, D. Lagares, and A.M. Tager. 2016. Fibrogenic lung injury induces non-cell-autonomous fibroblast invasion. Am. J. Respir. Cell Mol. Biol. 54:831–842. 10.1165/rcmb.2015-0040OC 26600305 PMC4942213

[bib3] Artaud-Macari, E., D. Goven, S. Brayer, A. Hamimi, V. Besnard, J. Marchal-Somme, Z.E. Ali, B. Crestani, S. Kerdine-Romer, A. Boutten, and M. Bonay. 2013. Nuclear factor erythroid 2-related factor 2 nuclear translocation induces myofibroblastic dedifferentiation in idiopathic pulmonary fibrosis. Antioxid. Redox Signal. 18:66–79. 10.1089/ars.2011.4240 22703534

[bib4] Baughman, R.P., E.E. Lower, M.A. Miller, P.A. Bejarano, and S.C. Heffelfinger. 1999. Overexpression of transforming growth factor-α and epidermal growth factor-receptor in idiopathic pulmonary fibrosis. Sarcoidosis Vasc. Diffuse Lung Dis. 16:57–61 10207942

[bib5] Black, M., D. Milewski, T. Le, X. Ren, Y. Xu, V.V. Kalinichenko, and T.V. Kalin. 2018. FOXF1 inhibits pulmonary fibrosis by preventing CDH2-CDH11 cadherin switch in myofibroblasts. Cell Rep. 23:442–458. 10.1016/j.celrep.2018.03.067 29642003 PMC5947867

[bib6] Bolte, C., H.M. Flood, X. Ren, S. Jagannathan, A. Barski, T.V. Kalin, and V.V. Kalinichenko. 2017. FOXF1 transcription factor promotes lung regeneration after partial pneumonectomy. Sci. Rep. 7:10690. 10.1038/s41598-017-11175-3 28878348 PMC5587533

[bib7] Chen, H., J. Qu, X. Huang, A. Kurundkar, L. Zhu, N. Yang, A. Venado, Q. Ding, G. Liu, V.B. Antony, . 2016. Mechanosensing by the α6-integrin confers an invasive fibroblast phenotype and mediates lung fibrosis. Nat. Commun. 7:12564. 10.1038/ncomms12564 27535718 PMC4992155

[bib8] Cho, H.Y., S.P.M. Reddy, M. Yamamoto, and S.R. Kleeberger. 2004. The transcription factor NRF2 protects against pulmonary fibrosis. FASEB J. 18:1258–1260. 10.1096/fj.03-1127fje 15208274

[bib9] Chuang-Tsai, S., T.H. Sisson, N. Hattori, C.G. Tsai, N.M. Subbotina, K.E. Hanson, and R.H. Simon. 2003. Reduction in fibrotic tissue formation in mice genetically deficient in plasminogen activator inhibitor-1. Am. J. Pathol. 163:445–452. 10.1016/S0002-9440(10)63674-7 12875966 PMC1868204

[bib10] Concepcion, C.P., S. Ma, L.M. LaFave, A. Bhutkar, M. Liu, L.P. DeAngelo, J.Y. Kim, I. Del Priore, A.J. Schoenfeld, M. Miller, . 2022. SMARCA4 inactivation promotes lineage-specific transformation and early metastatic features in the lung. Cancer Dis. 12:562–585. 10.1158/2159-8290.CD-21-0248 PMC8831463 34561242

[bib11] Epstein Shochet, G., E. Brook, O. Eyal, E. Edelstein, and D. Shitrit. 2019. Epidermal growth factor receptor paracrine upregulation in idiopathic pulmonary fibrosis fibroblasts is blocked by nintedanib. Am. J. Physiol. Lung Cell. Mol. Physiol. 316:L1025–L1034. 10.1152/ajplung.00526.2018 30810067

[bib12] Faress, J.A., D.E. Nethery, E.F.O. Kern, R. Eisenberg, F.J. Jacono, C.L. Allen, and J.A. Kern. 1985. Bleomycin-induced pulmonary fibrosis is attenuated by a monoclonal antibody targeting HER2. J. Appl. Physiol. 103:2077–2083. 10.1152/japplphysiol.00239.2007 17916677

[bib13] Geng, Y., X. Liu, J. Liang, D.M. Habiel, V. Kulur, A.L. Coelho, N. Deng, T. Xie, Y. Wang, N. Liu, . 2019. PD-L1 on invasive fibroblasts drives fibrosis in a humanized model of idiopathic pulmonary fibrosis. JCI Insight. 4:e125326. 10.1172/jci.insight.125326 PMC6482997 30763282

[bib14] Geyer, C.E., J. Forster, D. Lindquist, S. Chan, C.G. Romieu, T. Pienkowski, A. Jagiello-Gruszfeld, J. Crown, A. Chan, B. Kaufman, . 2006. Lapatinib plus capecitabine for HER2-positive advanced breast cancer. N. Engl. J. Med. 355:2733–2743. 10.1056/NEJMoa064320 17192538

[bib15] Granata, S., G. Santoro, V. Masola, P. Tomei, F. Sallustio, P. Pontrelli, M. Accetturo, N. Antonucci, P. Carratu, A. Lupo, and G. Zaza. 2018. In vitro identification of new transcriptomic and miRNomic profiles associated with pulmonary fibrosis induced by high doses everolimus: Looking for new pathogenetic markers and therapeutic targets. Int. J. Mol. Sci. 19:1250. 10.3390/ijms19041250 PMC5979287 29677166

[bib16] Habermann, A.C., A.J. Gutierrez, L.T. Bui, S.L. Yahn, N.I. Winters, C.L. Calvi, L. Peter, M.I. Chung, C.J. Taylor, C. Jetter, . 2020. Single-cell RNA sequencing reveals profibrotic roles of distinct epithelial and mesenchymal lineages in pulmonary fibrosis. Sci. Adv. 6:eaba1972. 10.1126/sciadv.aba1972 32832598 PMC7439444

[bib17] Habiel, D.M., M.S. Espindola, A.L. Coelho, and C.M. Hogaboam. 2018. Modeling idiopathic pulmonary fibrosis in humanized severe combined immunodeficient mice. Am. J. Pathol. 188:891–903. 10.1016/j.ajpath.2017.12.020 29378172 PMC5954978

[bib18] Hecker, L., N.J. Logsdon, D. Kurundkar, A. Kurundkar, K. Bernard, T. Hock, E. Meldrum, Y.Y. Sanders, and V.J. Thannickal. 2014. Reversal of persistent fibrosis in aging by targeting Nox4-Nrf2 redox imbalance. Sci. Transl. Med. 6:231ra47. 10.1126/scitranslmed.3008182 PMC4545252 24718857

[bib19] Huan, C., T. Yang, J. Liang, T. Xie, L. Cheng, N. Liu, A. Kurkciyan, J. Monterrosa Mena, C. Wang, H. Dai, . 2015. Methylation-mediated BMPER expression in fibroblast activation in vitro and lung fibrosis in mice in vivo. Sci. Rep. 5:14910. 10.1038/srep14910 26442443 PMC4595647

[bib20] Huang, S.K., A.M. Scruggs, R.C. McEachin, E.S. White, and M. Peters-Golden. 2014. Lung fibroblasts from patients with idiopathic pulmonary fibrosis exhibit genome-wide differences in DNA methylation compared to fibroblasts from nonfibrotic lung. PLoS One. 9:e107055. 10.1371/journal.pone.0107055 25215577 PMC4162578

[bib21] Imokawa, S., A. Sato, H. Hayakawa, M. Kotani, T. Urano, and A. Takada. 1997. Tissue factor expression and fibrin deposition in the lungs of patients with idiopathic pulmonary fibrosis and systemic sclerosis. Am. J. Respir. Crit. Care Med. 156:631–636. 10.1164/ajrccm.156.2.9608094 9279250

[bib22] Kang, H.R., C.G. Lee, R.J. Homer, and J.A. Elias. 2007. Semaphorin 7A plays a critical role in TGF-β1-induced pulmonary fibrosis. J. Exp. Med. 204:1083–1093. 10.1084/jem.20061273 17485510 PMC2118575

[bib23] King, T.E., Jr., W.Z. Bradford, S. Castro-Bernardini, E.A. Fagan, I. Glaspole, M.K. Glassberg, E. Gorina, P.M. Hopkins, D. Kardatzke, L. Lancaster, . 2014. A phase 3 trial of pirfenidone in patients with idiopathic pulmonary fibrosis. N. Engl. J. Med. 370:2083–2092. 10.1056/NEJMoa1402582 24836312

[bib24] Kramer, A., J. Green, J. Pollard Jr., and S. Tugendreich. 2014. Causal analysis approaches in ingenuity pathway analysis. Bioinformatics. 30:523–530. 10.1093/bioinformatics/btt703 24336805 PMC3928520

[bib25] Kuhn, C., 3rd, J. Boldt, T.E. King Jr, E. Crouch, T. Vartio, and J.A. McDonald. 1989. An immunohistochemical study of architectural remodeling and connective tissue synthesis in pulmonary fibrosis. Am. Rev. Respir. Dis. 140:1693–1703. 10.1164/ajrccm/140.6.1693 2604297

[bib26] Li, Y., D. Jiang, J. Liang, E.B. Meltzer, A. Gray, R. Miura, L. Wogensen, Y. Yamaguchi, and P.W. Noble. 2011. Severe lung fibrosis requires an invasive fibroblast phenotype regulated by hyaluronan and CD44. J. Exp. Med. 208:1459–1471. 10.1084/jem.20102510 21708929 PMC3135364

[bib27] Liu, X., S.C. Rowan, J. Liang, C. Yao, G. Huang, N. Deng, T. Xie, D. Wu, Y. Wang, A. Burman, . 2021. Categorization of lung mesenchymal cells in development and fibrosis. iScience. 24:102551. 10.1016/j.isci.2021.102551 34151224 PMC8188567

[bib28] Loskutoff, D.J., and J.P. Quigley. 2000. PAI-1, fibrosis, and the elusive provisional fibrin matrix. J. Clin. Invest. 106:1441–1443. 10.1172/JCI11765 11120750 PMC381477

[bib29] Lovgren, A.K., J.J. Kovacs, T. Xie, E.N. Potts, Y. Li, W.M. Foster, J. Liang, E.B. Meltzer, D. Jiang, R.J. Lefkowitz, and P.W. Noble. 2011. β-arrestin deficiency protects against pulmonary fibrosis in mice and prevents fibroblast invasion of extracellular matrix. Sci. Transl. Med. 3:74ra23. 10.1126/scitranslmed.3001564 PMC3094726 21411739

[bib30] Luhtala, N., and R. Parker. 2009. LSM1 over-expression in *Saccharomyces cerevisiae* depletes U6 snRNA levels. Nucleic Acids Res. 37:5529–5536. 10.1093/nar/gkp572 19596813 PMC2760792

[bib31] Mackman, N. 2004. Role of tissue factor in hemostasis, thrombosis, and vascular development. Arterioscler. Thromb. Vasc. Biol. 24:1015–1022. 10.1161/01.ATV.0000130465.23430.74 15117736

[bib32] Mahlapuu, M., S. Enerback, and P. Carlsson. 2001. Haploinsufficiency of the forkhead gene Foxf1, a target for sonic hedgehog signaling, causes lung and foregut malformations. Development. 128:2397–2406. 10.1242/dev.128.12.2397 11493558

[bib33] Martinelli, M., A.M.G. Pacilli, S. Rivetti, M. Lauriola, L. Fasano, P. Carbonara, G. Mattei, I. Valentini, L. Scapoli, and R. Solmi. 2011. A role for epidermal growth factor receptor in idiopathic pulmonary fibrosis onset. Mol. Biol. Rep. 38:4613–4617. 10.1007/s11033-010-0594-0 21132379

[bib34] Melino, G. 2011. p63 is a suppressor of tumorigenesis and metastasis interacting with mutant p53. Cell Death Differ. 18:1487–1499. 10.1038/cdd.2011.81 21760596 PMC3178431

[bib35] Morse, C., T. Tabib, J. Sembrat, K.L. Buschur, H.T. Bittar, E. Valenzi, Y. Jiang, D.J. Kass, K. Gibson, W. Chen, . 2019. Proliferating SPP1/MERTK-expressing macrophages in idiopathic pulmonary fibrosis. Eur. Respir. J. 54:1802441. 10.1183/13993003.02441-2018 31221805 PMC8025672

[bib36] Ng, B., J. Dong, G. D’Agostino, S. Viswanathan, A.A. Widjaja, W.W. Lim, N.S.J. Ko, J. Tan, S.P. Chothani, B. Huang, . 2019. Interleukin-11 is a therapeutic target in idiopathic pulmonary fibrosis. Sci. Transl. Med. 11:eaaw1237. 10.1126/scitranslmed.aaw1237 31554736

[bib37] Noble, P.W. 2006. Idiopathic pulmonary fibrosis: Natural history and prognosis. Clin. Chest Med. 27:S11–S16, v. 10.1016/j.ccm.2005.08.003 16545628

[bib38] Noble, P.W., C.E. Barkauskas, and D. Jiang. 2012. Pulmonary fibrosis: Patterns and perpetrators. J. Clin. Invest. 122:2756–2762. 10.1172/JCI60323 22850886 PMC3408732

[bib39] Oh, D.Y., and Y.J. Bang. 2020. HER2-targeted therapies: A role beyond breast cancer. Nat. Rev. Clin. Oncol. 17:33–48. 10.1038/s41571-019-0268-3 31548601

[bib40] Olman, M.A., N. Mackman, C.L. Gladson, K.M. Moser, and D.J. Loskutoff. 1995. Changes in procoagulant and fibrinolytic gene expression during bleomycin-induced lung injury in the mouse. J. Clin. Invest. 96:1621–1630. 10.1172/JCI118201 7544811 PMC185788

[bib41] Paliogiannis, P., S.S. Fois, A.G. Fois, A. Cossu, G. Palmieri, and G. Pintus. 2021. Repurposing anticancer drugs for the treatment of idiopathic pulmonary fibrosis and antifibrotic drugs for the treatment of cancer: State of the art. Curr. Med. Chem. 28:2234–2247. 10.2174/0929867327999200730173748 32748739

[bib42] Pandit, K.V., D. Corcoran, H. Yousef, M. Yarlagadda, A. Tzouvelekis, K.F. Gibson, K. Konishi, S.A. Yousem, M. Singh, D. Handley, . 2010. Inhibition and role of let-7d in idiopathic pulmonary fibrosis. Am. J. Respir. Critical Care Med. 182:220–229. 10.1164/rccm.200911-1698OC 20395557 PMC2913236

[bib43] Pardo, A., and M. Selman. 2012. Role of matrix metaloproteases in idiopathic pulmonary fibrosis. Fibrogenesis Tissue Repair. 5:S9. 10.1186/1755-1536-5-S1-S9 23259796 PMC3368759

[bib44] Park, J., D.S. Kim, T.S. Shim, C.M. Lim, Y. Koh, S.D. Lee, W.S. Kim, W.D. Kim, J.S. Lee, and K.S. Song. 2001. Lung cancer in patients with idiopathic pulmonary fibrosis. Eur. Respir. J. 17:1216–1219. 10.1183/09031936.01.99055301 11491167

[bib45] Pierce, E.M., K. Carpenter, C. Jakubzick, S.L. Kunkel, K.R. Flaherty, F.J. Martinez, and C.M. Hogaboam. 2007. Therapeutic targeting of CC ligand 21 or CC chemokine receptor 7 abrogates pulmonary fibrosis induced by the adoptive transfer of human pulmonary fibroblasts to immunodeficient mice. Am. J. Pathol. 170:1152–1164. 10.2353/ajpath.2007.060649 17392156 PMC1829450

[bib46] Pondé, N., M. Brandao, G. El-Hachem, E. Werbrouck, and M. Piccart. 2018. Treatment of advanced HER2-positive breast cancer: 2018 and beyond. Cancer Treat Rev. 67:10–20. 10.1016/j.ctrv.2018.04.016 29751334

[bib47] Powell, E., D. Piwnica-Worms, and H. Piwnica-Worms. 2014. Contribution of p53 to metastasis. Cancer Dis. 4:405–414. 10.1158/2159-8290.CD-13-0136 PMC4063123 24658082

[bib48] Qian, F., D.L. Vaux, and I.L. Weissman. 1994. Expression of the integrin α-4-β-1 on melanoma-cells can inhibit the invasive stage of metastasis formation. Cell. 77:335–347. 10.1016/0092-8674(94)90149-x 8181055

[bib49] Richeldi, L., R.M. du Bois, G. Raghu, A. Azuma, K.K. Brown, U. Costabel, V. Cottin, K.R. Flaherty, D.M. Hansell, Y. Inoue, . 2014. Efficacy and safety of nintedanib in idiopathic pulmonary fibrosis. N. Engl. J. Med. 370:2071–2082. 10.1056/NEJMoa1402584 24836310

[bib50] Robichaux, J.P., X. Le, R.S.K. Vijayan, J.K. Hicks, S. Heeke, Y.Y. Elamin, H.Y. Lin, H. Udagawa, F. Skoulidis, H. Tran, . 2021. Structure-based classification predicts drug response in EGFR-mutant NSCLC. Nature. 597:732–737. 10.1038/s41586-021-03898-1 34526717 PMC8481125

[bib51] Song, X., W. Liu, S. Xie, M. Wang, G. Cao, C. Mao, and C. Lv. 2013. All-transretinoic acid ameliorates bleomycin-induced lung fibrosis by downregulating the TGF-β1/Smad3 signaling pathway in rats. Lab. Invest. 93:1219–1231. 10.1038/labinvest.2013.108 24042439

[bib52] Stern, H.M. 2012. Improving treatment of HER2-positive cancers: Opportunities and challenges. Sci. Transl. Med. 4:127rv2. 10.1126/scitranslmed.3001539 22461643

[bib53] Trujillo, G., A. Meneghin, K.R. Flaherty, L.M. Sholl, J.L. Myers, E.A. Kazerooni, B.H. Gross, S.R. Oak, A.L. Coelho, H. Evanoff, . 2010. TLR9 differentiates rapidly from slowly progressing forms of idiopathic pulmonary fibrosis. Sci. Transl. Med. 2:57ra82. 10.1126/scitranslmed.3001510 PMC3235647 21068441

[bib54] Tsukui, T., K.H. Sun, J.B. Wetter, J.R. Wilson-Kanamori, L.A. Hazelwood, N.C. Henderson, T.S. Adams, J.C. Schupp, S.D. Poli, I.O. Rosas, . 2020. Collagen-producing lung cell atlas identifies multiple subsets with distinct localization and relevance to fibrosis. Nat. Commun. 11:1920. 10.1038/s41467-020-15647-5 32317643 PMC7174390

[bib55] Tzouvelekis, A., P. Spagnolo, F. Bonella, C. Vancheri, V. Tzilas, B. Crestani, M. Kreuter, and D. Bouros. 2018. Patients with IPF and lung cancer: Diagnosis and management. Lancet Respir. Med. 6:86–88. 10.1016/S2213-2600(17)30478-2 29241977

[bib56] Vancheri, C. 2013. Common pathways in idiopathic pulmonary fibrosis and cancer. Eur. Respir. Rev. 22:265–272. 10.1183/09059180.00003613 23997054 PMC9487347

[bib57] Wajant, H., K. Pfizenmaier, and P. Scheurich. 2003. Tumor necrosis factor signaling. Cell Death Differ. 10:45–65. 10.1038/sj.cdd.4401189 12655295

[bib58] Wang, Y.C., J.S. Liu, H.K. Tang, J. Nie, J.X. Zhu, L.L. Wen, and Q.L. Guo. 2016. miR-221 targets HMGA2 to inhibit bleomycin-induced pulmonary fibrosis by regulating TGF-β1/Smad3-induced EMT. Int. J. Mol. Med. 38:1208–1216. 10.3892/ijmm.2016.2705 27513632

[bib59] Xie, T., J. Liang, N. Liu, C. Huan, Y. Zhang, W. Liu, M. Kumar, R. Xiao, J. D’Armiento, D. Metzger, . 2016. Transcription factor TBX4 regulates myofibroblast accumulation and lung fibrosis. J. Clin. Investig. 126:3626–3679. 10.1172/JCI89968 PMC5004952 27548522

[bib60] Xie, T., Y. Wang, N. Deng, G. Huang, F. Taghavifar, Y. Geng, N. Liu, V. Kulur, C. Yao, P. Chen, . 2018. Single-cell deconvolution of fibroblast heterogeneity in mouse pulmonary fibrosis. Cell Rep. 22:3063–3640. 10.1016/j.celrep.2018.03.010 PMC5908225 29590628

[bib61] Yang, Y.M., M. Noureddin, C. Liu, K. Ohashi, S.Y. Kim, D. Ramnath, E.E. Powell, M.J. Sweet, Y.S. Roh, I.F. Hsin, . 2019. Hyaluronan synthase 2-mediated hyaluronan production mediates Notch1 activation and liver fibrosis. Sci. Transl. Med. 11:eaat9284. 10.1126/scitranslmed.aat9284 31189722 PMC6589184

[bib62] Zheng, G.X.Y., J.M. Terry, P. Belgrader, P. Ryvkin, Z.W. Bent, R. Wilson, S.B. Ziraldo, T.D. Wheeler, G.P. McDermott, J. Zhu, . 2017. Massively parallel digital transcriptional profiling of single cells. Nat. Commun. 8:14049. 10.1038/ncomms14049 28091601 PMC5241818

[bib63] Zucker, S.N., E.E. Fink, A. Bagati, S. Mannava, A. Bianchi-Smiraglia, P.N. Bogner, J.A. Wawrzyniak, C. Foley, K.I. Leonova, M.J. Grimm, . 2014. Nrf2 amplifies oxidative stress via induction of Klf9. Mol. Cell. 53:916–928. 10.1016/j.molcel.2014.01.033 24613345 PMC4049522

