# Supplementary material for: HER2 drives lung fibrosis by activating a metastatic cancer signature in invasive lung fibroblasts
Source: J Exp Med. 2022 Aug 18;219(10):e20220126. doi: 10.1084/jem.20220126 (PMC9391950; doi:10.1084/jem.20220126)
Supplement: Table S1 — shows human sample donor information and summary of scRNA-seq experiments. [file JEM_20220126_TableS1.docx]

| **Table. S1 Human sample donor information and summary of scRNA-seq experiments.** | | | | | | | | | |
| --- | --- | --- | --- | --- | --- | --- | --- | --- | --- |
| This table provides the human sample donor information and the number of individual invasive and non-invasive lung fibroblasts profiled in each IPF or CTL sample before quality control (QC) and after QC. | | | | | | | | | |
| Donor information of the samples in scRNA-seq | | | | | | | | | |
| Sample ID | Patient information | | | Cell number (Before QC) | | | Cell number (After QC) | | |
|  | Age | Gender | Disease | invasive | non-invasive | Total | invasive | non-invasive | Total |
| CTL1 | 17 | M | normal | 4,096 | 4,450 | 8,546 | 3,966 | 4,401 | 8,367 |
| CTL2 | 21 | M | normal | 5,310 | 4,636 | 9,946 | 4,778 | 4,007 | 8,785 |
| CTL3 | 51 | F | normal | 7,701 | 2,918 | 10,619 | 6,357 | 2,340 | 8,697 |
| CTL4 | 52 | M | normal | 2,930 | 3,402 | 6,332 | 2,911 | 3,368 | 6,279 |
| IPF1 | 72 | M | IPF | 4,812 | 4,565 | 9,377 | 4,760 | 4,555 | 9,315 |
| IPF2 | 59 | M | IPF | 5,280 | 4,739 | 10,019 | 4,094 | 3,270 | 7,364 |
| IPF3 | 71 | M | IPF | 5,910 | 5,175 | 11,085 | 4,586 | 3,238 | 7,824 |
| IPF4 | 73 | M | IPF | 3,093 | 3,801 | 6,894 | 3,067 | 3,780 | 6,847 |
| Donor information of the samples in Fig 5E and G | | | | | | | | | |
| Fig 5E | Age | Gender | Disease | Part | Fig 5G | Age | Gender | Disease | Part |
| sample 1 | na | na | normal | na | sample 1 | 78 | M | normal | na |
| sample 2 | 2 | M | normal | na | sample 2 | 18 | M | normal | na |
| sample 3 | 78 | M | normal | na | sample 3 | 78 | F | normal | D+P |
| sample 4 | 18 | M | normal | na | sample 4 | 55 | F | IPF | na |
| sample 5 | 71 | M | IPF | D+P | sample 5 | na | na | IPF | na |
| sample 6 | na | na | IPF | D+P | sample 6 | 58 | M | IPF | na |
| sample 7 | 65 | M | IPF | na | sample 7 | 71 | M | IPF | na |
| sample 8 | 58 | M | IPF | D+P | na, not available  D+P, distal and proximal | | | | |
| sample 9 | 55 | F | IPF | na |  |  |  |  |  |
