# Supplementary material for: HER2 drives lung fibrosis by activating a metastatic cancer signature in invasive lung fibroblasts
Source: J Exp Med. 2022 Aug 18;219(10):e20220126. doi: 10.1084/jem.20220126 (PMC9391950; doi:10.1084/jem.20220126)
Supplement: Table S2 — lists primer sequences for qRT-PCR. [file JEM_20220126_TableS2.docx]

| **Table. S2 Primer sequences for qRT-PCR** | | |
| --- | --- | --- |
| Gene | Primer sequence | |
|  | Forward (5' -> 3’) | Reverse (5' -> 3’) |
| *SEMA7A* | TGTGTATTCCCTCGGTGACA | GATCTCCATCATGTTGAAGG |
| *F3* | TCCCCAGAGTTCACACCTTACCT | CACTTTTGTTCCCACCTGTTCA |
| *ITGA6* | GAGCTTTTGTGATGGGCGATT | CTCTCCACCAACTTCATAAGGC |
| *FOXF1* | GCGGCTTCCGAAGGAAATG | CAAGTGGCCGTTCATCATGC |
| *CREBRF* | GCCATCTGAGTGGAATCGAGA | CCGTGACTTCTTAACTGCGTATT |
| *TSC22D1* | CTGACGACACCCCTGGTGGAT | CGATTTTGTTGTCAATAGCTACCACAC |
| *MXI1* | CAACGTGCAGCGTCTGCTGGAGGC | CGATTCTTTTCCAGCTCATTGTG |
| *KLF9* | TGGCTGTGGGAAAGTCTATGG | CTCGTCTGAGCGGGAGAACT |
| *NFE2L2* | AGCCCAGCACATCCAGTCA | TGCATGCAGTCATCAAAGTACAAAG |
| *HMGA2* | AAAGCAGCTCAAAAGAAAGCA | TGTTGTGGCCATTTCCTAGGT |
| *DPF3* | GGCTGCTGGAGATAAAACCTGA | TTCCTGGATGCTTTCCTCCTC |
| *ERBB2* (*HER2*) | CCAGCTGGCTCTCACACTG | AGCCCTTACACATCGGAGAAC |
| *FENDRR* | AATTGCTGGGCTGCTTTCTA | TTCACAATGGCTCAGTGCTC |
| *LINC00152* | TCTTCACAGCACAGTTCCTGG | GGCTGAGTCGTGATTTTCGG |
