# Supplementary figures and images for: HER2 drives lung fibrosis by activating a metastatic cancer signature in invasive lung fibroblasts
Source: J Exp Med. 2022 Aug 18;219(10):e20220126. doi: 10.1084/jem.20220126 (PMC9391950; doi:10.1084/jem.20220126)

Figure 1E

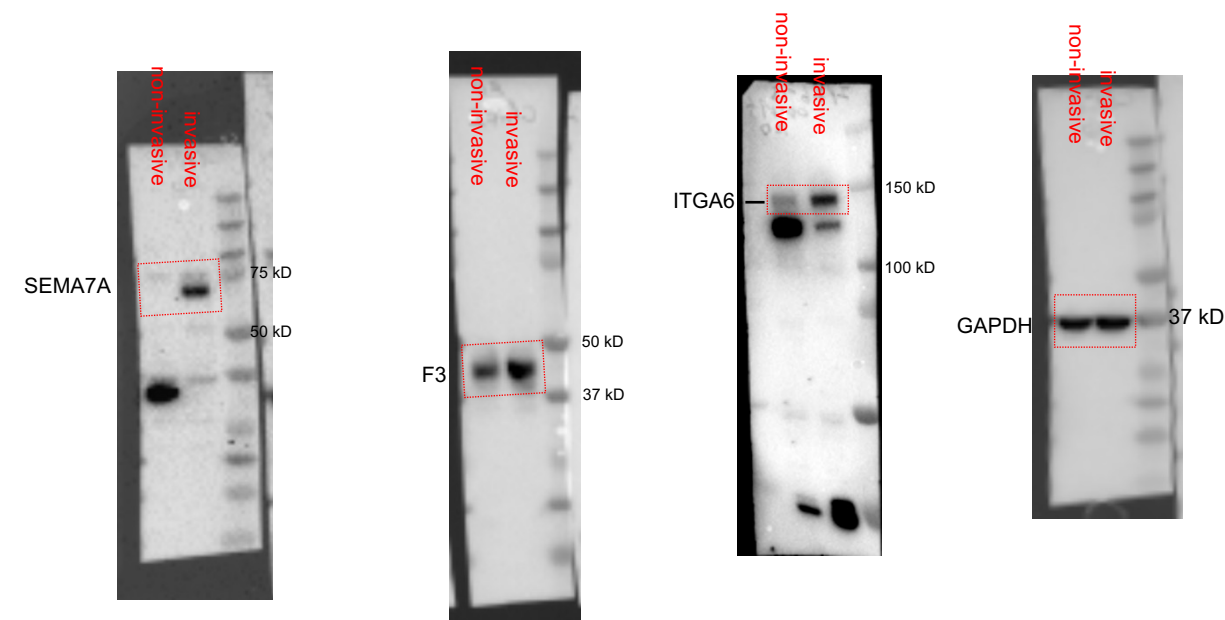

Supplement: SourceData F1 — contains original blots for Fig. 1. [file JEM_20220126_SourceDataF1.pdf]

Figure 2C

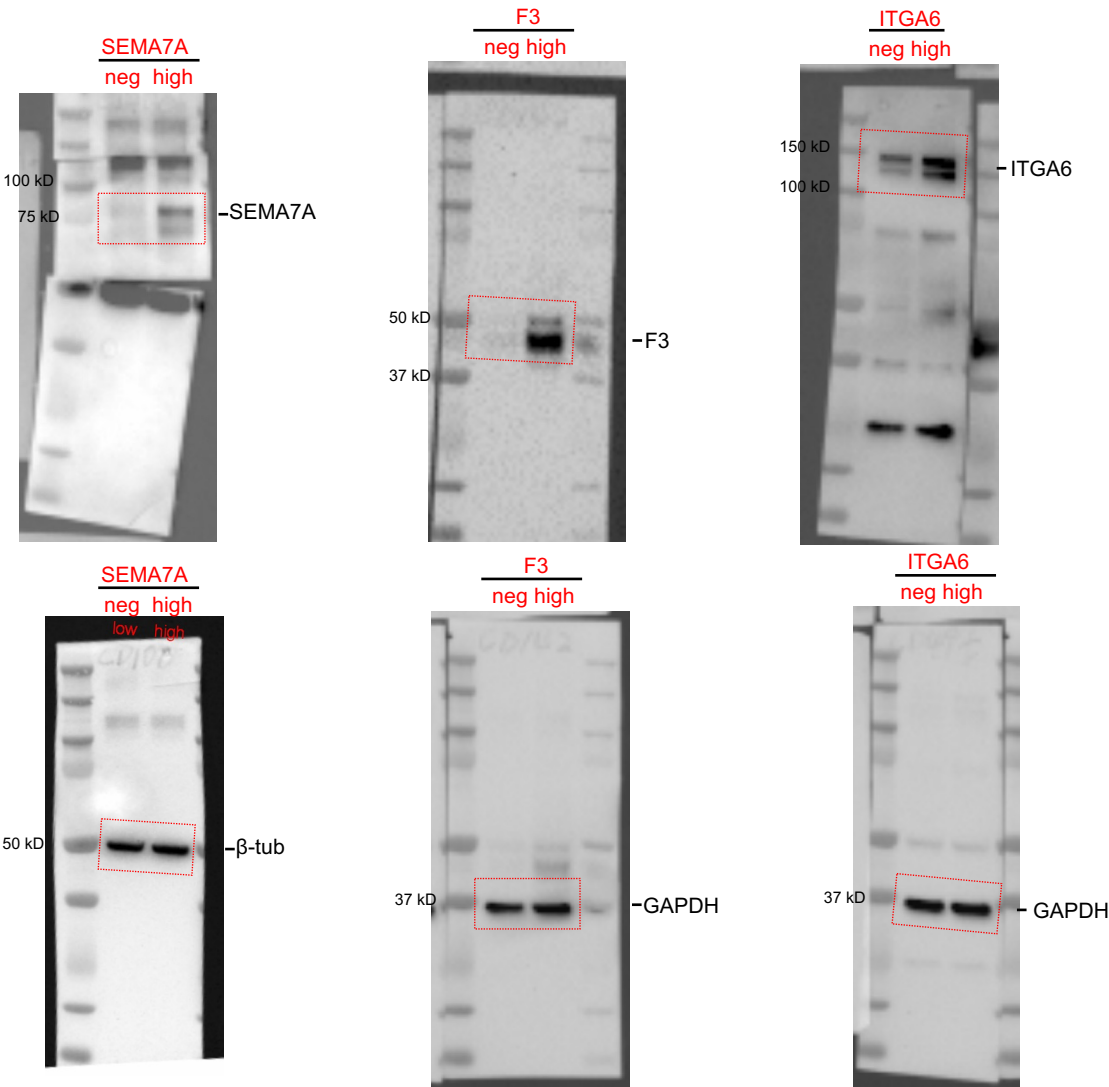

Figure 2G

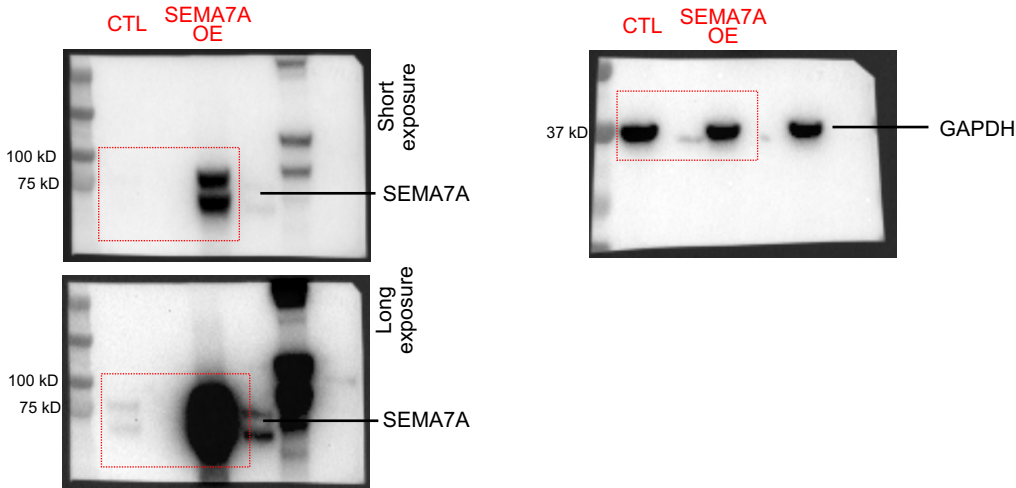

Supplement: SourceData F2 — contains original blots for Fig. 2. [file JEM_20220126_SourceDataF2.pdf]

Figure 3E-F

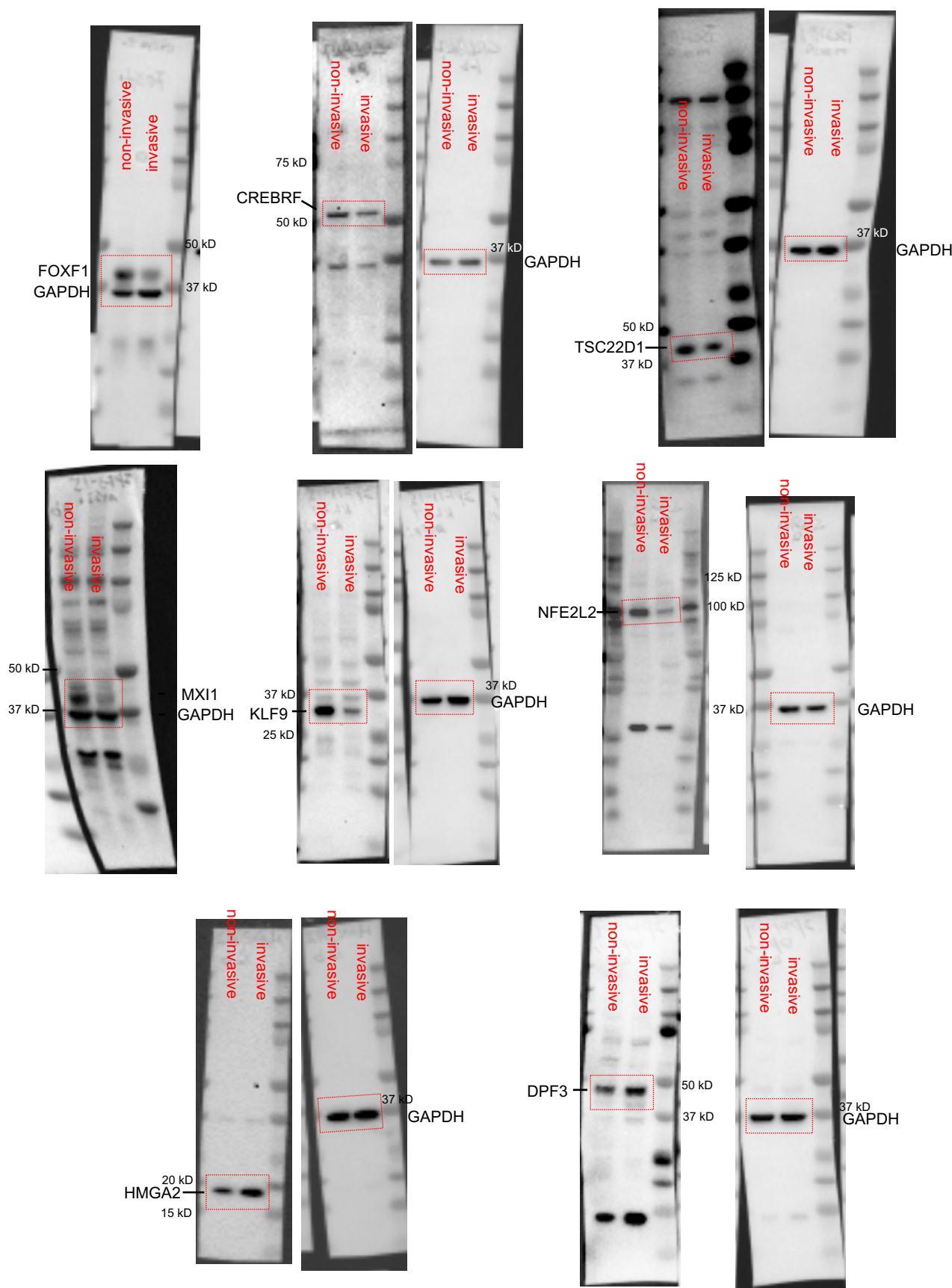

Supplement: SourceData F3 — contains original blots for Fig. 3. [file JEM_20220126_SourceDataF3.pdf]

Figure 4C-D

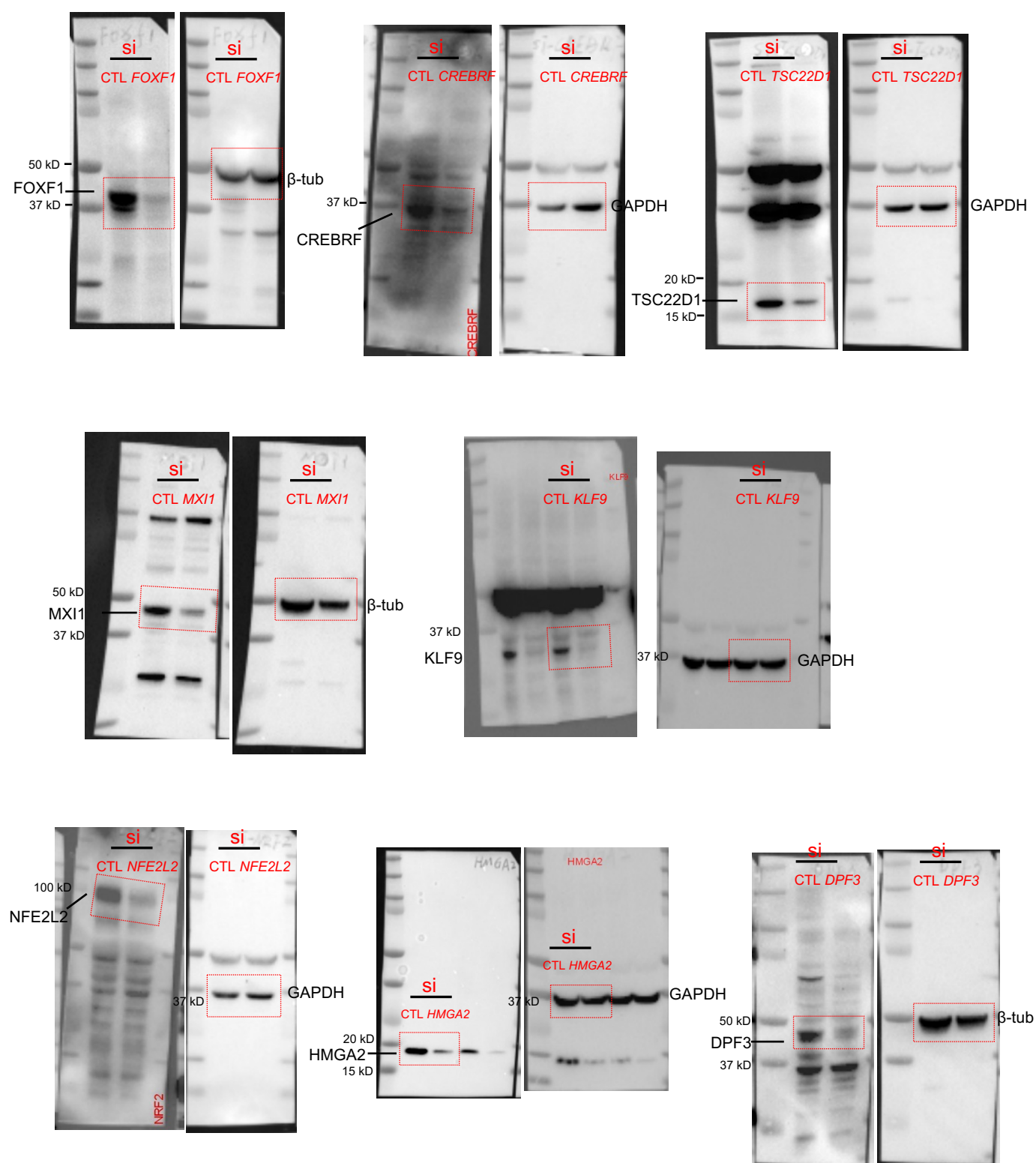

Supplement: SourceData F4 — contains original blots for Fig. 4. [file JEM_20220126_SourceDataF4.pdf]

Figure 5E

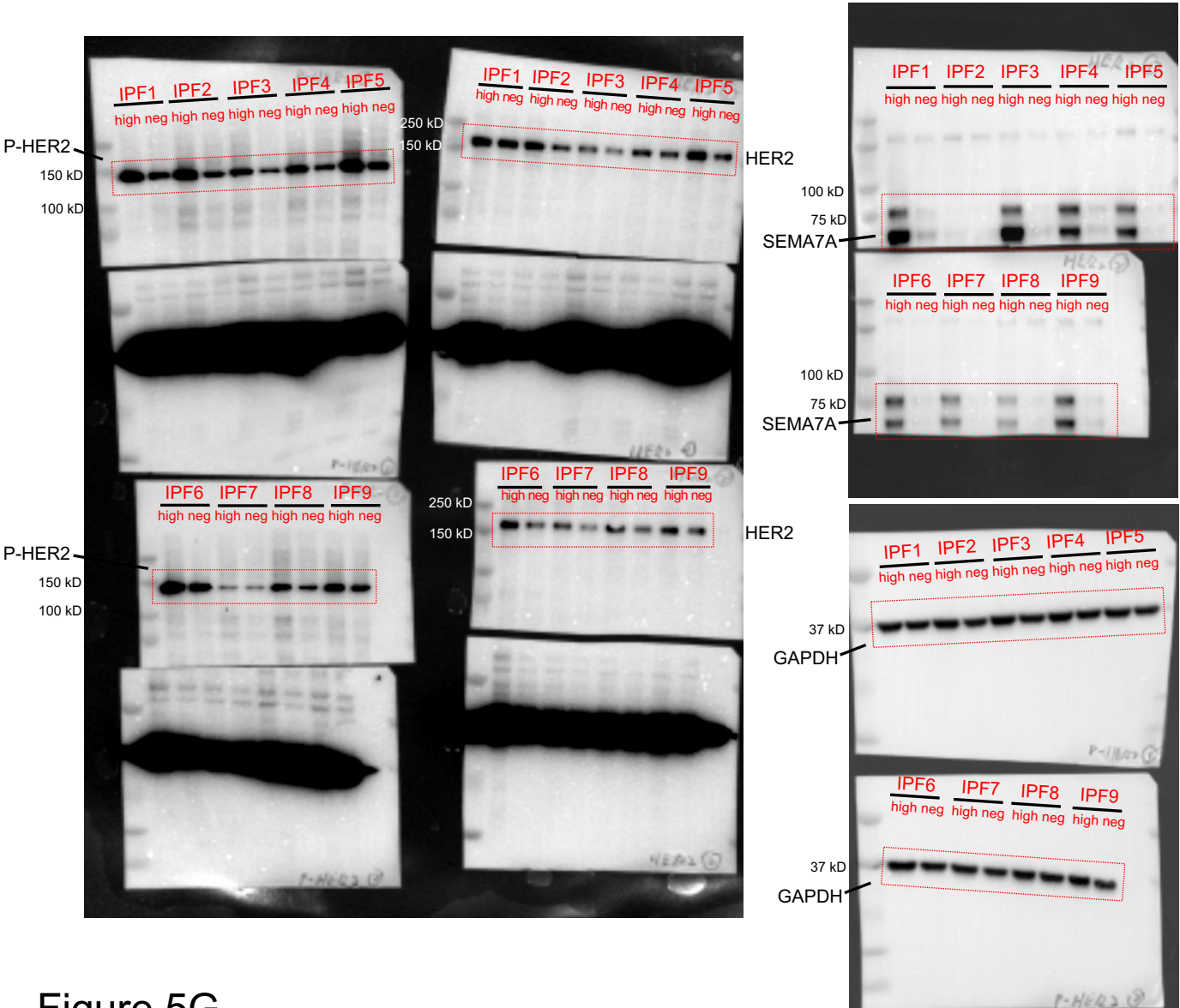

Figure 5G

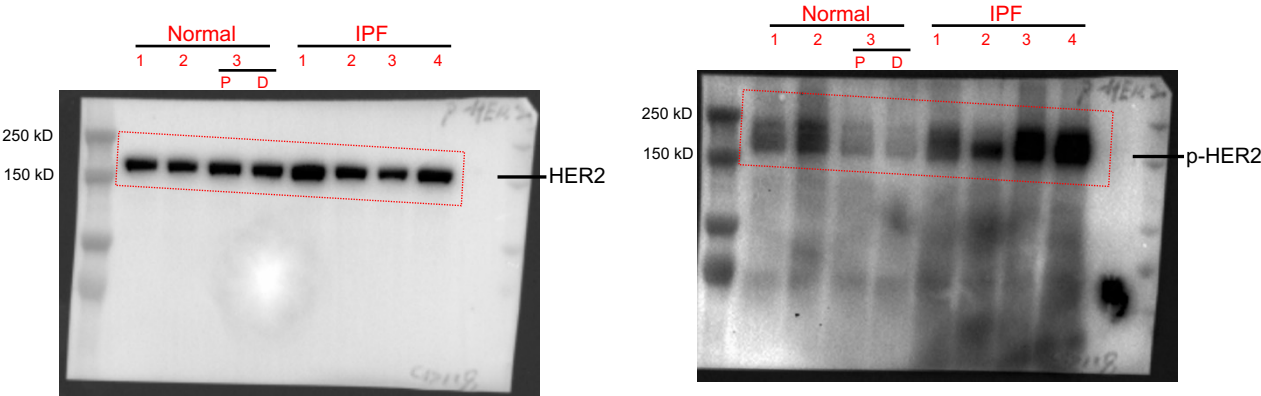

Supplement: SourceData F5 — contains original blots for Fig. 5. [file JEM_20220126_SourceDataF5.pdf]

### Figure 6F

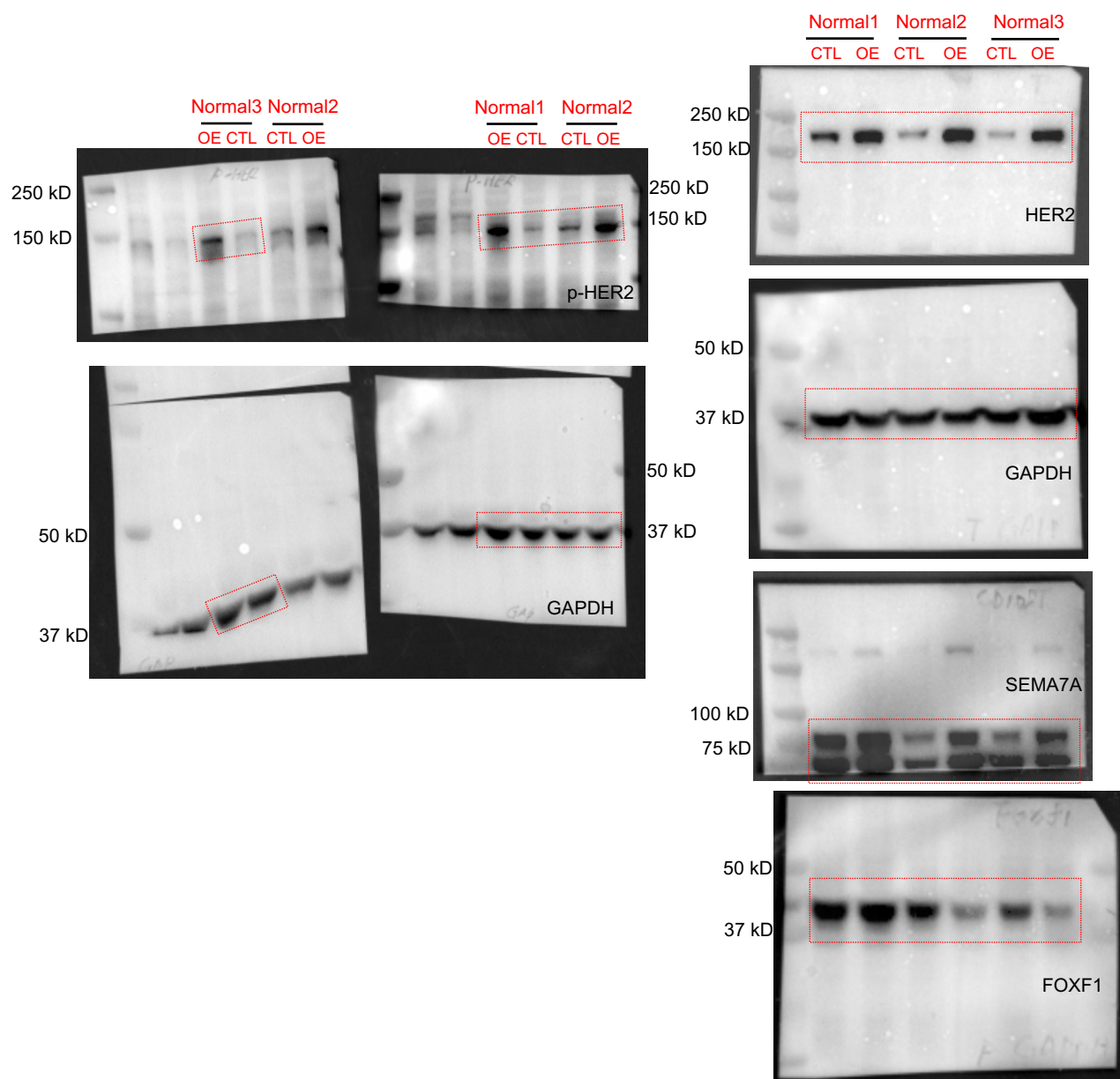

Supplement: SourceData F6 — contains original blots for Fig. 6. [file JEM_20220126_SourceDataF6.pdf]

Figure 7B

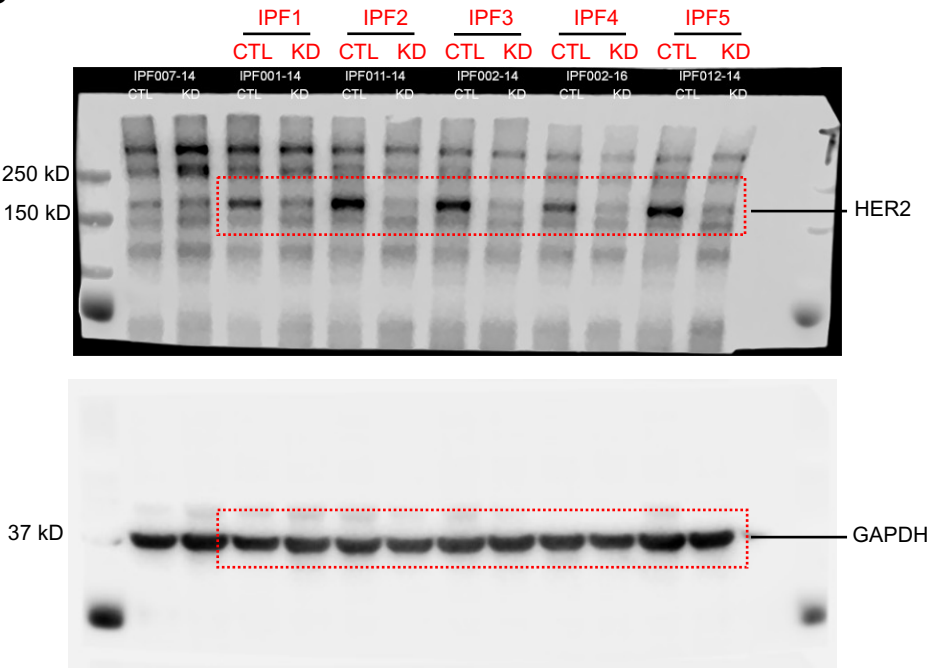

Figure 7G

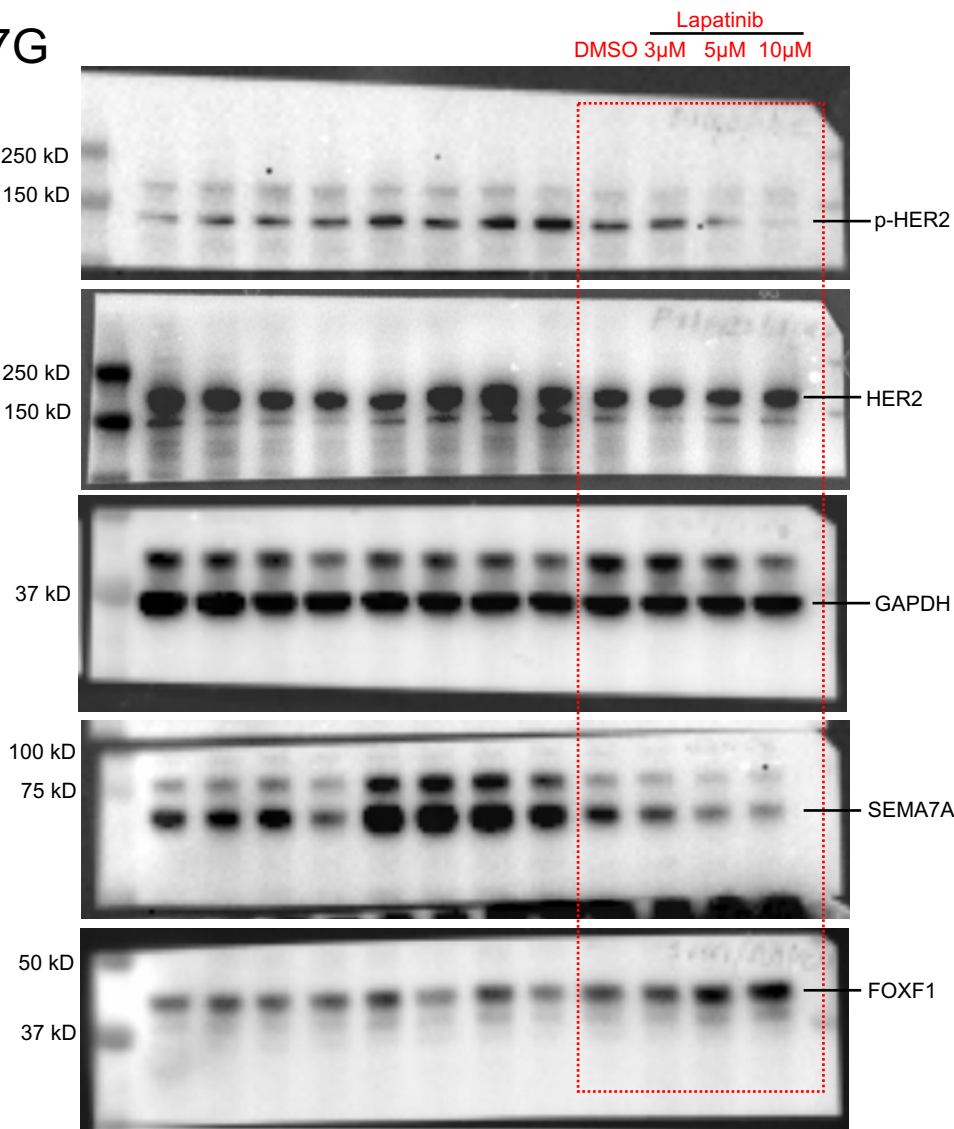

Supplement: SourceData F7 — contains original blots for Fig. 7. [file JEM_20220126_SourceDataF7.pdf]
